# Hypusination Orchestrates the Antimicrobial Response of Macrophages

**DOI:** 10.1016/j.celrep.2020.108510

**Published:** 2020-12-15

**Authors:** Alain P. Gobert, Jordan L. Finley, Yvonne L. Latour, Mohammad Asim, Thaddeus M. Smith, Thomas G. Verriere, Daniel P. Barry, Margaret M. Allaman, Alberto G. Delagado, Kristie L. Rose, M. Wade Calcutt, Kevin L. Schey, Johanna C. Sierra, M. Blanca Piazuelo, Raghavendra G. Mirmira, Keith T. Wilson

**Affiliations:** 1 Division of Gastroenterology, Hepatology, and Nutrition, Department of Medicine, Vanderbilt University Medical Center, Nashville, TN 37232, USA; 2 Center for Mucosal Inflammation and Cancer, Vanderbilt University Medical Center, Nashville, TN 37232, USA; 3 Department of Pathology, Microbiology, and Immunology, Vanderbilt University School of Medicine, Nashville, TN 37232, USA; 4 Department of Biochemistry, Mass Spectrometry Research Center, Vanderbilt University School of Medicine, Nashville, TN 37232, USA; 5 Translational Research Center, Department of Medicine, The University of Chicago, Chicago, IL 60637, USA; 6 Veterans Affairs Tennessee Valley Healthcare System, Nashville, TN 37232, USA; 7 Lead Contact

## Abstract

Innate responses of myeloid cells defend against pathogenic bacteria via inducible effectors. Deoxyhypusine synthase (DHPS) catalyzes the transfer of the N-moiety of spermidine to the lysine-50 residue of eukaryotic translation initiation factor 5A (EIF5A) to form the amino acid hypusine. Hypusinated EIF5A (EIF5A^Hyp^) transports specific mRNAs to ribosomes for translation. We show that DHPS is induced in macrophages by two gastrointestinal pathogens, *Helicobacter pylori* and *Citrobacter rodentium*, resulting in enhanced hypusination of EIF5A. EIF5A^Hyp^ was also increased in gastric macrophages from patients with *H. pylori* gastritis. Furthermore, we identify the bacteria-induced immune effectors regulated by hypusination. This set of proteins includes essential constituents of antimicrobial response and autophagy. Mice with myeloid cell-specific deletion of *Dhps* exhibit reduced EIF5A^Hyp^ in macrophages and increased bacterial burden and inflammation. Thus, regulation of translation through hypusination is a critical hallmark of the defense of eukaryotic hosts against pathogenic bacteria.

## In Brief

Gobert et al. demonstrate that hypusination, a specific mechanism regulating translation, is induced in macrophages by bacteria. Hypusination is required for the translation of inducible antimicrobial effectors. Mice that specifically lack hypusination in macrophages are highly susceptible to *Helicobacter pylori* and *Citrobacter rodentium*, two pathogens of the gastrointestinal tract.

## Graphical Abstract

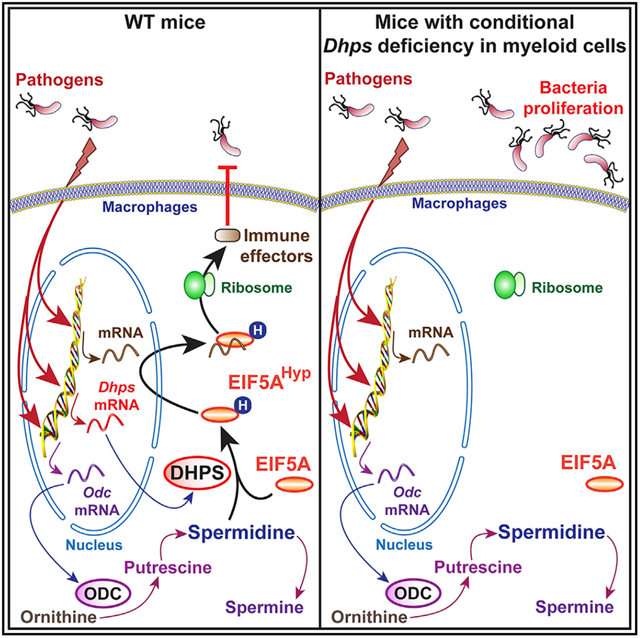

## INTRODUCTION

The mammalian gastrointestinal tract is the home of the largest population of myeloid cells (Lee et al., 1985). These resident and infiltrating cells respond to inflammatory signals and to foreign antigens, thus exhibiting enhanced antimicrobial abilities that are needed for the clearance of pathogens (Hardbower et al., 2017; Niess et al., 2005; Wennerås et al., 2000). In this context, deciphering the molecular mechanisms that regulate the innate response of these immune cells is essential for a better understanding of the protective immunity against gastrointestinal pathogens.

The arginase-ornithine decarboxylase (ODC) metabolic pathway plays an essential role in the orchestration of the activation of macrophages (Latour et al., 2020). Arginase activity converts arginine into urea and ornithine, with the latter being metabolized by ODC into putrescine (Pegg, 2009). This first and shortest polyamine is then sequentially converted by spermidine synthase and spermine synthase into spermidine and spermine, respectively (Latour et al., 2020; Pegg, 2016). We have implicated putrescine in the pathophysiology of infection with *Helicobacter pylori* (Hardbower et al., 2017), a specific pathogen of the stomach responsible for diseases ranging from gastritis to gastric cancer (Peek et al., 2010). Using mice with myeloid-specific *Odc* knockout, we found that endogenous putrescine alters histone methylation/acetylation and dampens the transcription of the genes encoding proinflammatory markers (Hardbower et al., 2017). This results in the limitation of inflammation and the exacerbation of *H. pylori* burden (Hardbower et al., 2017). In addition, we have established that spermine reduces arginine uptake, which is needed for nitric oxide (NO) synthase 2 (NOS2) translation and NO-dependent killing of *H. pylori*, thus impairing immunity to the bacterium (Bussière et al., 2005; Chaturvedi et al., 2010). However, the importance of spermidine for the outcome of the infection remains unknown.

**Figure 1.**
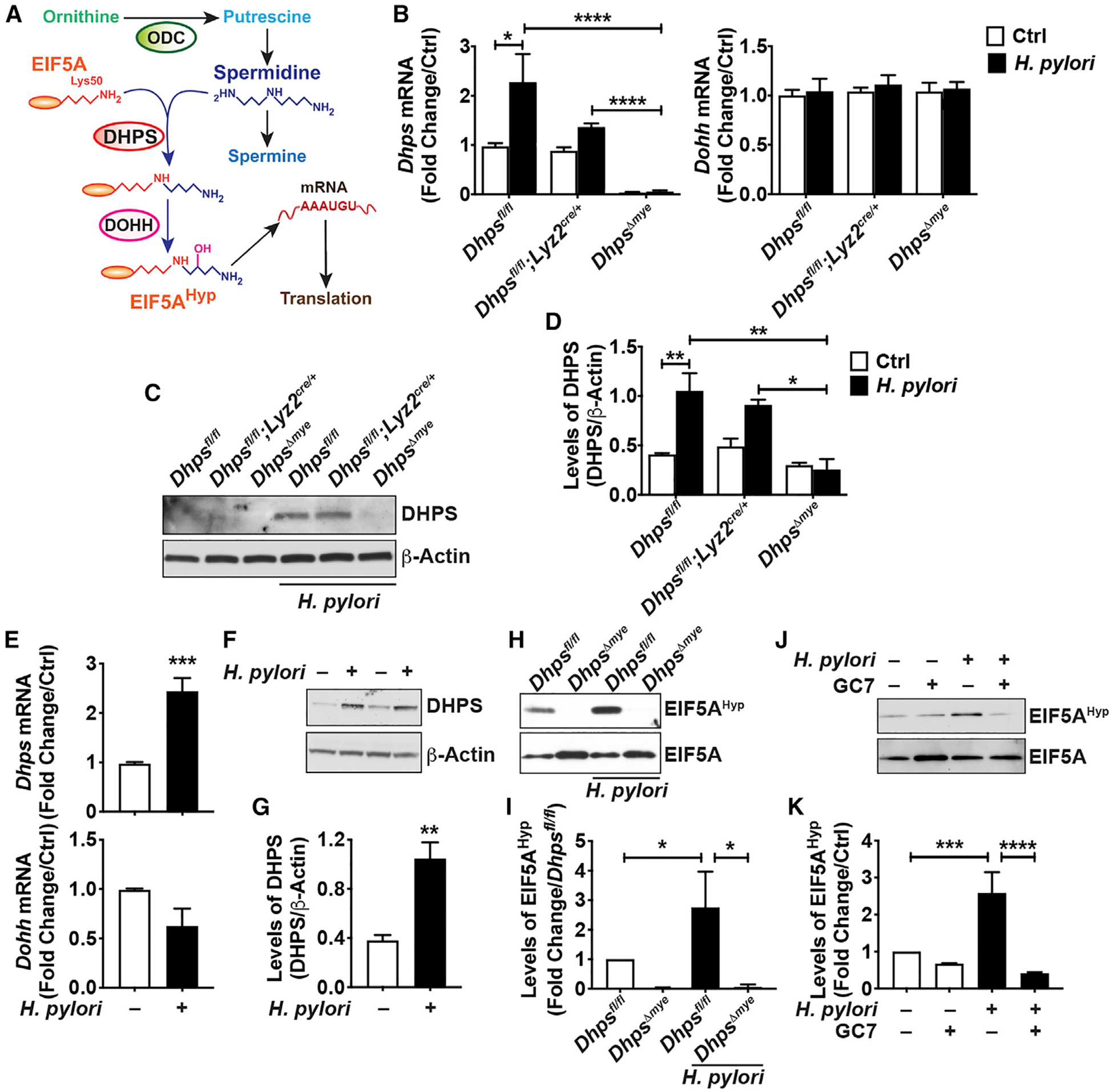
DHPS Expression and Activity in Macrophages (A) Schematic representation of the synthesis and role of EIF5A^Hyp^. (B) mRNA expression of *Dhps* and *Dohh* in BMmacs from *Dhps*^*fl/fl*^, *Dhps*^*fl/fl*^*;Lyz2*^*cre/+*^, and *Dhps*^*Δmye*^ mice stimulated or not with *H. pylori* (*Hp*) PMSS1 for 6 h; *n* = 3 mice per genotype. (C and D) DHPS protein levels were assessed by western blot in the same cells infected or not for 24 h (C); representative data of macrophages isolated from three different mice per genotype. The densitometric analysis of DHPS was performed from the three animals/group (D). (E) *Dhps* and *Dohh* mRNA levels in RAW 264.7 cells infected or not with *Hp* for 6 h; *n* = 7 independent experiments. (F and G) Representative immunoblot for DHPS using protein lysates from RAW 264.7 cells ± *Hp* (F); densitometry from five independent western blots is shown in (G). (H–K) Levels of EIF5A^Hyp^ and EIF5A in *Dhps*^*fl/fl*^- and *Dhps*^*Δmye*^-derived BMmacs (H and I) and in RAW 264.7 macrophages (J and K) infected or not with *Hp* for 24 h. The densitometry was determined from three immunoblots for RAW 264.7 cells and BMmacs. For all the panels, each bar represents the mean ± SEM; *p < 0.05, **p < 0.01, ***p < 0.001, and ****p < 0.0001. See also Figures S1–S3.

Spermidine has been shown to improve cardiac function, to be neuroprotective, and to dampen inflammation (Madeo et al., 2018), notably by stimulating autophagy and mitophagy (Eisenberg et al., 2009, 2016; Qi et al., 2016). Importantly, spermidine is required for the synthesis of the non-proteinogenic amino acid hypusine. The only known protein that contains hypusine is the eukaryotic translation initiation factor 5A (EIF5A; Park et al., 1981; Park and Wolff, 2018). Two isoforms of EIF5A exist: EIF5A1, referred to as eIF5A in this study, which is expressed universally; and eIF5A2, which is mainly present in testes, brain, and tumor cells (Jenkins et al., 2001; Nakanishi and Cleveland, 2016). Hypusine is formed by the action of two enzymes: deoxyhypusine synthase (DHPS), which transfers the N-terminal moiety of spermidine to the lysine-50 residue in EIF5A; and deoxyhypusine hydroxylase (DOHH) that completes hypusine biosynthesis by hydroxylation of the side chain (Figure 1A; Joe et al., 1995; Park et al., 2006). This post-translational modification of EIF5A, called hypusination, has been reported to be essential for two molecular events. On the one hand, EIF5A^Hyp^ binds specific mRNAs that contain a 5′-AAAUGU-3′ consensus sequence (Maier et al., 2010; Xu and Chen, 2001; Xu et al., 2004), and the EIF5A^Hyp^/mRNA complex is then translocated to the cytoplasm by the nuclear transporter exportin-1 (Aksu et al., 2016), reaches ribosomes, and allows for translation (Park and Wolff, 2018). On the other hand, hypusinated EIF5A alleviates ribosome pausing during the translation of peptides enriched in diprolyl, diglycyl motifs and in basic and acidic amino acids (Gutierrez et al., 2013; Pelechano and Alepuz, 2017; Schuller et al., 2017). Hypusination is crucial for embryonic development, cell differentiation, and proliferation (Ganapathi et al., 2019; Nishimura et al., 2005; Park et al., 1997; Sievert et al., 2014). When small interfering RNA (siRNA) against EIF5A or mice with specific deletion of *Dhps* in pancreatic islet β cells were used, it was shown that hypusinated EIF5A leads to the development of glucose intolerance in inflammatory mouse models of diabetes, notably by supporting NOS2 translation (Levasseur et al., 2019; Maier et al., 2010). In addition, hypusination in macrophages supports the tricarboxylic acid (TCA) cycle and oxidative phosphorylation by promoting the translation of mitochondrial proteins and promotes alternative (M2) activation (Puleston et al., 2019). However, the role of hypusination in the innate response of macrophages during bacterial infection has not been determined. We hypothesized that protein translation by the DHPS/EIF5A^Hyp^ pathway is critical for the appropriate response of macrophages to invaders.

In this report, we show that the enzyme DHPS is induced by *H. pylori* in macrophages. This results in increased hypusination of EIF5A *in vitro* and in mouse and human tissues with *H. pylori* gastritis. Using mice with specific deletion of *Dhps* in myeloid cells, and macrophages treated with the DHPS inhibitor *N*^1^-guanyl-1,7-diaminoheptane (GC7), we identified a series of inducible proteins regulated by hypusination in infected macrophages. These effectors are notably involved in the antimicrobial response and autophagy. Deletion of DHPS in myeloid cells resulted in higher survival of phagocytized bacteria and increased colonization *in vivo* by the gastric pathogen *H. pylori* and by the colonic pathogen *Citrobacter rodentium*, the rodent equivalent of the human pathogen enteropathogenic *Escherichia coli*. Thus, in addition to the classical transcriptional response of macrophages, we emphasize herein that the regulation of translation through hypusination is a critical hallmark of the defense of the mammalian host against pathogenic bacteria.

## RESULTS

### Pathogenic Bacteria Stimulate DHPS Induction

We used the *Lyz2*-*Cre* driver to generate mice with specific deletion of *Dhps* in the myeloid cell lineage, including macrophages, dendritic cells, and granulocytes. Based on the conceptual framework of the role of hypusination in innate immunity, we first assessed the macrophage response to *H. pylori* infection. In response to this pathogen, bone-marrow-derived macrophages (BMmacs) from *Dhps*^*fl/fl*^ mice exhibited a significant increase in *Dhps* mRNA expression, whereas the *Dohh* level was not affected by the infection (Figure 1B). The expression of *Dhps* in control or infected macrophages from *Dhps*^*fl/fl*^*;Lyz2*^*cre/cre*^ mice (hereafter referred to as *Dhps*^*Δmye*^ mice) was significantly reduced compared with cells from *Dhps*^*fl/fl*^ or *Dhps*^*fl/fl*^*;Lyz2*^*cre/+*^ mice (Figure 1B). Similarly, the *H. pylori*-induced DHPS protein expression in BMmacs from *Dhps*^*fl/fl*^ and *Dhps*^*fl/fl*^*;Lyz2*^*cre/+*^ mice was markedly attenuated in *Dhps*^*Δmye*^ cells (Figure 1C). By densitometry, we found that the level of DHPS was reduced in *H. pylori*-stimulated macrophages from *Dhps*^*Δmye*^ mice to the same level as unstimulated controls (Figure 1D). In addition, the level of *Dhps* mRNA, but not *Dohh* mRNA (Figure 1E), as well as DHPS protein (Figures 1F and 1G), was increased in the murine macrophage cell line RAW 264.7 infected with *H. pylori*. This induction was reduced by blocking phosphatidylinositol 3-kinase (PI3K) with LY294002 (Figures S1A and S1B); however, inhibitors of nuclear factor κB (NF-κB), mitogen-activated protein kinase 14 (MAPK14), MAPK1/3, or MAP2K1 had no effect on *H. pylori*-induced DHPS protein expression (Figures S1A and S1B), indicating that signaling through PI3K is involved in stimulation of DHPS in infected macrophages. In addition, expression of *Dhps* mRNA (Figure S1C) and DHPS protein (Figure S1D) was also induced by the colonic pathogen *C. rodentium* in BMmacs from *Dhps*^*fl/fl*^ and *Dhps*^*fl/fl*^*;Lyz2*^*cre/+*^ mice and markedly reduced in *Dhps*^*Δmye*^ BMmacs. Lastly, we observed that DHPS was also induced by *H. pylori* and *C. rodentium* in bone-marrow-derived dendritic cells (BMDCs; Figure S1E).

Using a specific antibody (Nishiki et al., 2013), we found that the level of EIF5A^Hyp^ was enhanced in BMmacs from *Dhps*^*fl/fl*^ mice infected with *H. pylori* compared with uninfected cells and was absent in uninfected and infected *Dhps*^*Δmye*^ macrophages (Figures 1H and 1I). Similarly, *H. pylori*-induced hypusination in RAW 264.7 cells was significantly reduced by the DHPS inhibitor GC7 (Figures 1J and 1K). Moreover, we found hypusination of EIF5A was significantly induced by *H. pylori* in BMmacs from *Odc*^*fl/fl*^ mice and from animals with specific *Odc* deletion in myeloid cells (*Odc*^*Δmye*^; Hardbower et al., 2017; Figures S1F and S1G). While EIF5A^Hyp^ was reduced in BMmacs from *Odc*^*Δmye*^ mice compared with *Odc*^*fl/fl*^ mice, more than 50% of the EIF5A^Hyp^ remained in infected cells (Figures S1F and S1G), explaining why increased immune effectors could still be identified in BMmacs from *Odc*^*Δmye*^ mice lacking the transcriptional inhibitor putrescine (Hardbower et al., 2017).

These data demonstrate that bacterial infection of macrophages leads to enhanced hypusination, notably through the inducible expression of DHPS.

### Increased Hypusination in *H. pylori*-Infected Patients

**Figure 2.**
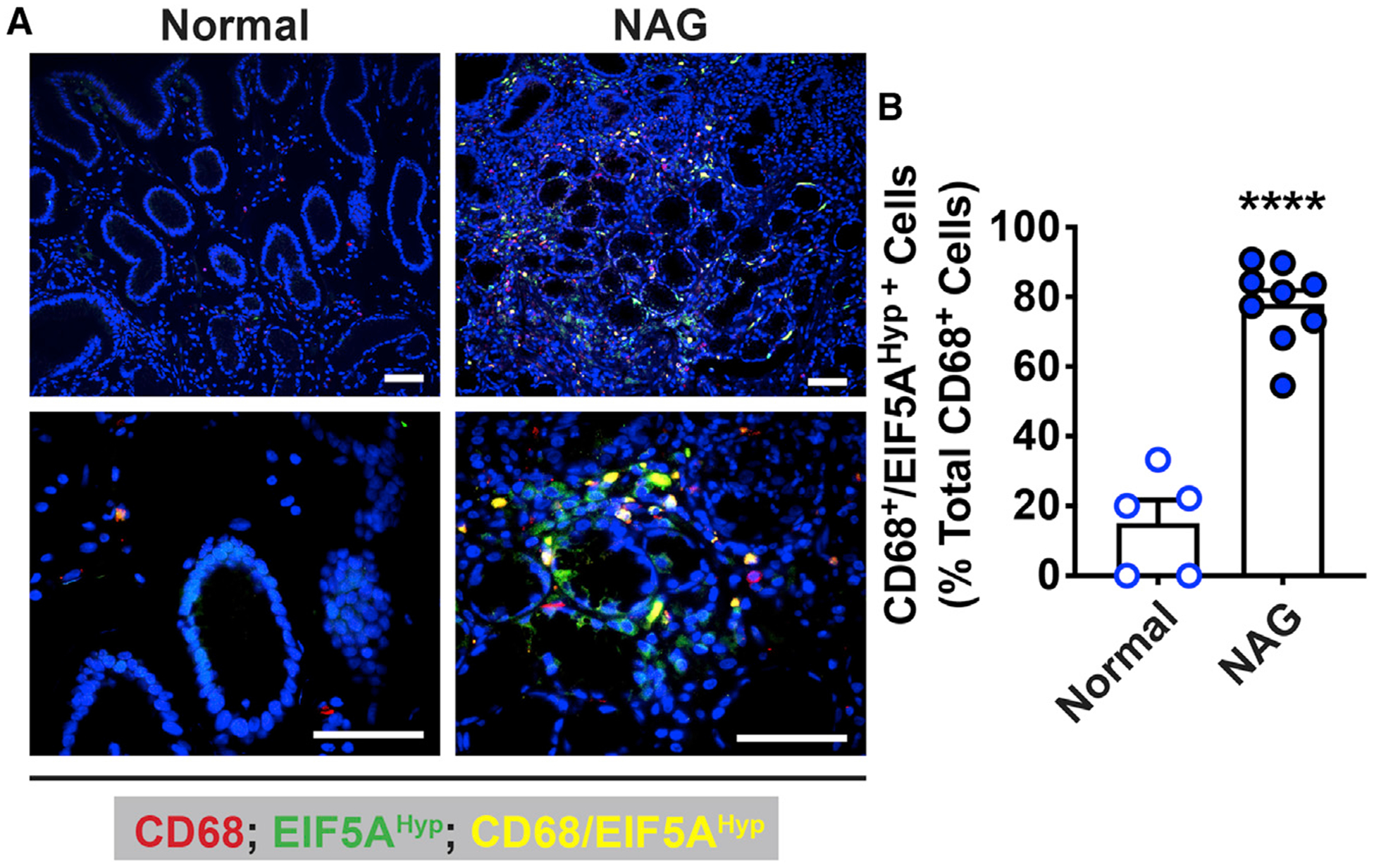
Levels of Hypusinated EIF5A in Human Gastric Tissues during *Hp* Infection (A) The macrophage marker CD68 (red), EIF5A^Hyp^ (green), and nuclei (blue) were detected by immunofluorescence in patients with normal histology and with non-atrophic gastritis (NAG) caused by infection. Merged images are shown, with cells double positive for CD68 and EIF5A^Hyp^ depicted in yellow. Scale bars, 50 μm. These photomicrographs are representative images for five normal and nine infected patients. (B) Quantification of EIF5A^Hyp^ staining in CD68^+^ cells. Bars represent mean ± SEM; ****p < 0.0001.

To determine whether DHPS is expressed in gastric macrophages in humans, we assessed gastric biopsies from uninfected individuals and *H. pylori*-infected patients with gastritis. The number of cells with double staining for EIF5A^Hyp^ and CD68 was significantly increased in tissues from infected patients compared with individuals without infection (Figures 2A and 2B).

### Hypusination Regulates Macrophage Function

**Figure 3.**
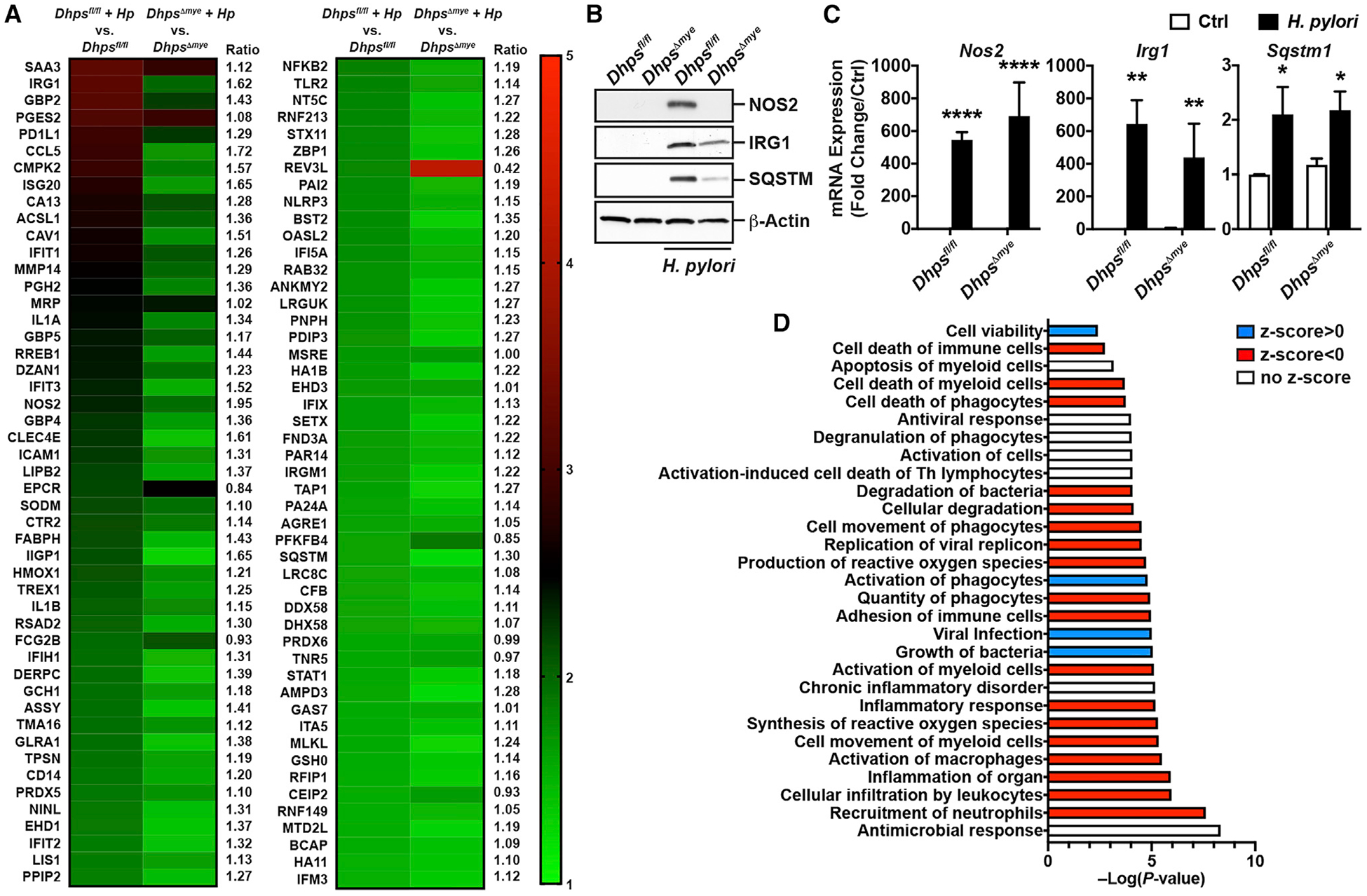
Regulation of the Inducible Proteome of Macrophages by *Dhps* Deletion (A) BMmacs from *Dhps*^*fl/fl*^ (*n* = 3) and *Dhps*^*Δmye*^ mice (*n* = 3) were infected with *Hp* PMSS1 for 24 h. Cells were lysed, and proteins were digested, labeled with iTRAQ reagents, and analyzed by liquid chromatography (LC)-coupled tandem mass spectrometry. The heatmap depicts the normalized fold change in the protein levels compared with uninfected cells; this panel represents the top 98 proteins induced by at least 1.3-fold in BMacs from *Dhps*^*fl/fl*^ and/or *Dhps*^*Δmye*^ mice. For each protein, the ratio between the fold increase in infected *Dhps*^*fl/fl*^ and *Dhps*^*Δmye*^ mice is indicated. (B) Levels of NOS2, IRG1, SQSTM, and β-actin proteins in BMmacs from *Dhps*^*fl/fl*^ and *Dhps*^*Δmye*^ mice infected or not with *Hp* for 24 h. (C) The expression of the genes encoding NOS2, IRG1, and SQSTM was analyzed in BMmacs infected or not for 6 h. In (B) and (C), *n* = 3–4 different mice per genotype. In all the panels, bars depict mean ± SEM; *p < 0.05, **p < 0.01, ***p < 0.001, and ****p < 0.0001. (D) IPA was performed on the proteome dataset obtained from BMmacs from *Dhps*^*fl/fl*^ and *Dhps*^*Δmye*^ mice, and the pathways related to diseases and functions are presented. The full lists of pathways are shown in Table S3. See also Figures S3–S5 and Tables S1, S2, and S3.

To determine the role of hypusination in the innate response of macrophages to bacterial infection, we analyzed the proteome of BMmacs from three different *Dhps*^*fl/fl*^ and *Dhps*^*Δmye*^ mice. These cells were stimulated or not with *H. pylori*, and an isobaric tag for relative and absolute quantification (iTRAQ)-based proteomics approach was utilized. We identified 2,994 proteins, comprising 2,895 murine proteins and 99 *H. pylori* proteins. There were 130 and 191 proteins induced by *H. pylori* infection in BMmacs from *Dhps*^*fl/fl*^ and *Dhps*^*Δmye*^ mice, respectively (Table S1). The most induced proteins in *Dhps*^*fl/fl*^ BMmacs included (1) classical antibacterial effectors IRG1, NOS2, and ASSY; (2) the proteins IFIH1, ISG20, GBP2/4, and IFIT2/3 that are involved in the antiviral response; (3) the proinflammatory cytokines IL1A, IL1B, and CCL5; (4) the inflammasome constituent NLRP3; and (5) the mediators of autophagy IIGP1, CLEC4E, and SQSTM (Figure 3A; Table S1). Remarkably, there were also proteins less expressed in infected BMmacs from *Dhps*^*Δmye*^ mice compared with *Dhps*^*fl/fl*^ animals; these included IRG1, NOS2, ASSY, IL1A, CCL5, IIGP1, CLEC4E, IRGM1, and SQSTM (Figure 3A; Table S1). We then used targeted approaches to verify our findings. We found by western blot that the level of the proteins NOS2, IRG1, and SQSTM were reduced in BMmacs from *Dhps*^*Δmye*^ mice infected with *H. pylori* compared with infected *Dhps*^*fl/fl*^-derived BMmacs (Figure 3B), thus validating the results obtained by proteomic analysis. We also confirmed that *de novo* translation of NOS2 was reduced in BMmacs from *Dhps*^*Δmye*^ mice compared with *Dhps*^*fl/fl*^ macrophages (Figure S2). The production of NO_2_^−^, the stable metabolite of NO derived from NOS2 activity, was also reduced in infected *Dhps*^*Δmye*^ BMmacs compared with cells from *Dhps*^*fl/fl*^ mice (Figure S3A). Importantly, the mRNA expression of the genes encoding these proteins was not affected by DHPS deletion (Figure 3C), further demonstrating that DHPS regulates only protein translation in infected macrophages. We also found that the expression of genes encoding for the chemokine CXCL1, the classical M1 marker IL-6, and the M2 marker arginase-1 was increased in response to *H. pylori* in both genotypes, but was not differentially expressed between them (Figure S3D), indicating that *Dhps* deletion in macrophages does not affect the inducible transcriptome in response to bacteria.

To confirm this first proteomic analysis, we performed a second iTRAQ experiment using a new set of BMmacs from one *Dhps*^*fl/fl*^ mouse and one *Dhps*^*Δmye*^ mouse. In this experiment, there were 248 proteins significantly upregulated by *H. pylori* in *Dhps*^*fl/fl*^ BMmacs (Table S2), and 78.5% of the induced proteins identified in the first experiment were also found in this independent study. Strikingly, the main *H. pylori*-stimulated immune mediators affected by *Dhps* deletion in the first experiment were also downregulated in BMmacs from *Dhps*^*Δmye*^ mice in this second experiment (Figure S4A). Additionally, we confirmed by immunoblotting that the immune effectors NOS2, IRG1, and SQSTM were induced in *Dhps*^*fl/fl*^ BMmacs infected with *C. rodentium* compared with control cells and that their expression was reduced in infected *Dhps*^*Δmye*^ BMmacs (Figure S4B), evidencing that the regulation of specific proteins by hypusination occurs in response to different pathogens. In this analysis, there were 27 and 13 proteins up- and downregulated, respectively, in uninfected *Dhps*^*Δmye*^ BMmacs compared with *Dhps*^*fl/fl*^ cells (Table S2), but there was no major regulator of the antimicrobial activity of macrophages in the uninfected cells.

The differential proteome dataset from *H. pylori*-infected BMmacs from *Dhps*^*fl/fl*^ and *Dhps*^*Δmye*^ mice was subjected to ingenuity pathway analysis (IPA) to determine biological functions and the important pathways regulated by hypusination in macrophages. The complete analysis is presented in Table S3. Strikingly, when we focused on the functions related to immune pathways, we found that the categories linked to macrophage activation and antimicrobial responses were significantly affected by *Dhps* deletion (Figure 3D); this was associated with an increase in “viral infection” and “growth of bacteria” pathways (Figure 3D). Overall, there was also a dampening of the pathways related to inflammation, adhesion, and death of immune cells (Figure 3D). In addition, we used IPA to identify the biochemical networks that were modulated in infected macrophages from *Dhps*^*Δmye*^ versus *Dhps*^*fl/fl*^ mice. The highest scored networks were “hematological system development and function, inflammatory response, tissue morphology” (score 29; Figure S5A) and “antimicrobial response, connective tissue disorders, inflammatory response” (score 26; Figure S5B), further indicating that the specific alterations in protein expression regulated by DHPS lead to enhanced host defenses.

**Figure 4.**
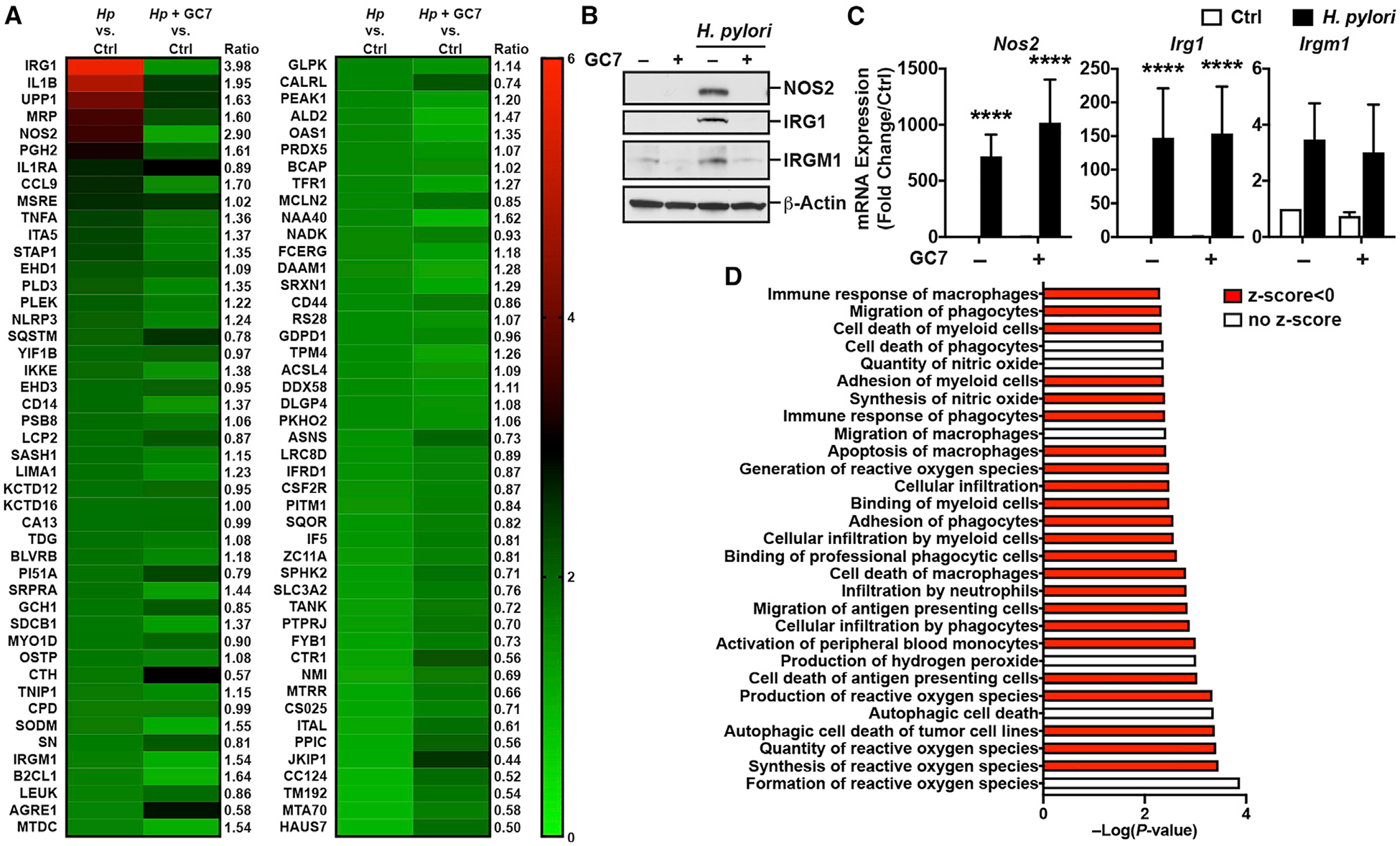
Effect of the Inhibition of DHPS on the Proteome of Activated Macrophages (A) Differential expression of proteins induced by *Hp* PMSS1 in RAW 264.7 cells ± GC7. The heatmap depicts the normalized fold change compared with uninfected cells of the 92 proteins induced significantly by at least 1.3-fold in infected RAW 264.7 macrophages, treated or not with GC7. For each protein, we calculated the ratio between the fold increase in infected macrophages and in infected macrophages + GC7. (B) Levels of NOS2, IRG1, IRGM1, and β-actin proteins in RAW 264.7 cells ± GC7 infected or not with *Hp* for 24 h; these immunoblots are representative data of three to six independent experiments. (C) Expression of the genes encoding NOS2, IRG1, and IRGM1 was determined in RAW 264.7 macrophages ± GC7 after a 6 h infection. Bars represent mean ± SEM; *p < 0.05, **p < 0.01, ***p < 0.001, and ****p < 0.0001; *n* = 3–6 independent experiments. (D) Diseases and functions pathways were determined by IPA using the differential proteome dataset obtained from RAW 264.7 cells. The full lists of pathways are shown in Table S5. See also Figure S5 and Tables S4 and S5.

Next, we assessed the effect of hypusination on the macrophage proteome using a pharmacological approach. RAW 264.7 cells were treated or not with the DHPS inhibitor GC7 for 2 h prior to infection with *H. pylori* PMSS1. After 24 h, the proteome was similarly analyzed using the iTRAQ-based experimental approach. We quantified 3,556 proteins, comprising 3,507 murine proteins and 49 *H. pylori* proteins. There were 57 and 58 proteins significantly upregulated in response to *H. pylori* in cells without and with GC7 (Table S4). We found a series of proteins more highly expressed in *H. pylori*-infected macrophages compared with infected cells treated with GC7, including many of the same proteins affected by *Dhps* deletion in BMmacs, e.g., IRG1, NOS2, EHD1, and IRGM1 (Figure 4A; Table S4). Of note, 58 proteins were induced by the GC7 in uninfected cells, whereas 158 were downregulated by DHPS inhibition; the proteins that were the most inhibited were ribosomal proteins (Table S4). We confirmed by immunoblotting that the proteins NOS2, IRG1, and IRGM1 were less induced in response to *H. pylori* in RAW 264.7 cells treated with GC7 compared with infected macrophages without GC7 (Figure 4B), while their mRNA levels were not affected (Figure 4C). In addition, the NO metabolite NO_2_^−^ was also produced in lower amounts by infected macrophages treated with GC7 (Figure S3B); GC7 also reduced *H. pylori*-induced autophagy in RAW 264.7 cells (Figure S3C). Lastly, the immune pathways involved in the activation and antimicrobial effects of macrophages were downregulated by GC7 in *H. pylori*-infected RAW 264.7 macrophages (Figure 4D; Table S5). Interestingly, the pathways predicted to impact cell death, including by autophagy, were also negatively affected by the inhibition of hypusination (Figure 4D; Table S5).

These data suggest that hypusination supports the antimicrobial protein expression profile of macrophages.

### mRNA and Protein Sequence Analysis of the Effectors Regulated by Hypusination in Macrophages

Hypusinated EIF5A binds preferentially to mRNAs that contains an 5′-AAAUGU-3′ consensus sequence (Maier et al., 2010; Xu and Chen, 2001). For this *in silico* analysis, we focused on the 48 proteins downregulated by at least 1.3-fold in infected *Dhps*^*Δmye*^ BMmacs and RAW 264.7 cells infected with *H. pylori* and treated with GC7. Among them, we found this binding site in the coding region of 23 of the proteins regulated by hypusination, and 20 more displayed the similar sequence with only one substitution (Table S6).

In addition, EIF5A^Hyp^ improves ribosome pausing during the translation of peptides enriched in diprolyl, diglycyl motifs and in basic and acidic amino acids (Gutierrez et al., 2013; Pelechano and Alepuz, 2017; Schuller et al., 2017). We identified 31 proteins with at least one diprolyl motif and 36 with one diglycyl motif (Table S6); 22 proteins exhibited at least one of each motif. Furthermore, we also identified 34 proteins containing at least one tripeptide whose translation is recognized to be facilitated by hypusinated EIF5A (Pelechano and Alepuz, 2017; Schuller et al., 2017). Only the proteins IFIT3, CCL5, and CLEC4E did not exhibit diprolyl, diglycyl, or EIF5A-regulated tripeptide motifs, but each of these did show the 5′-AAAUGU-3′ sequence (± 1 substitution).

Thus, all the innate effectors identified in this study to be affected by hypusination in macrophages exhibit at least one feature of EIF5A^Hyp^-regulated proteins, validating our proteomic findings.

### Regulation of the Mucosal Antimicrobial Response by DHPS

**Figure 5.**
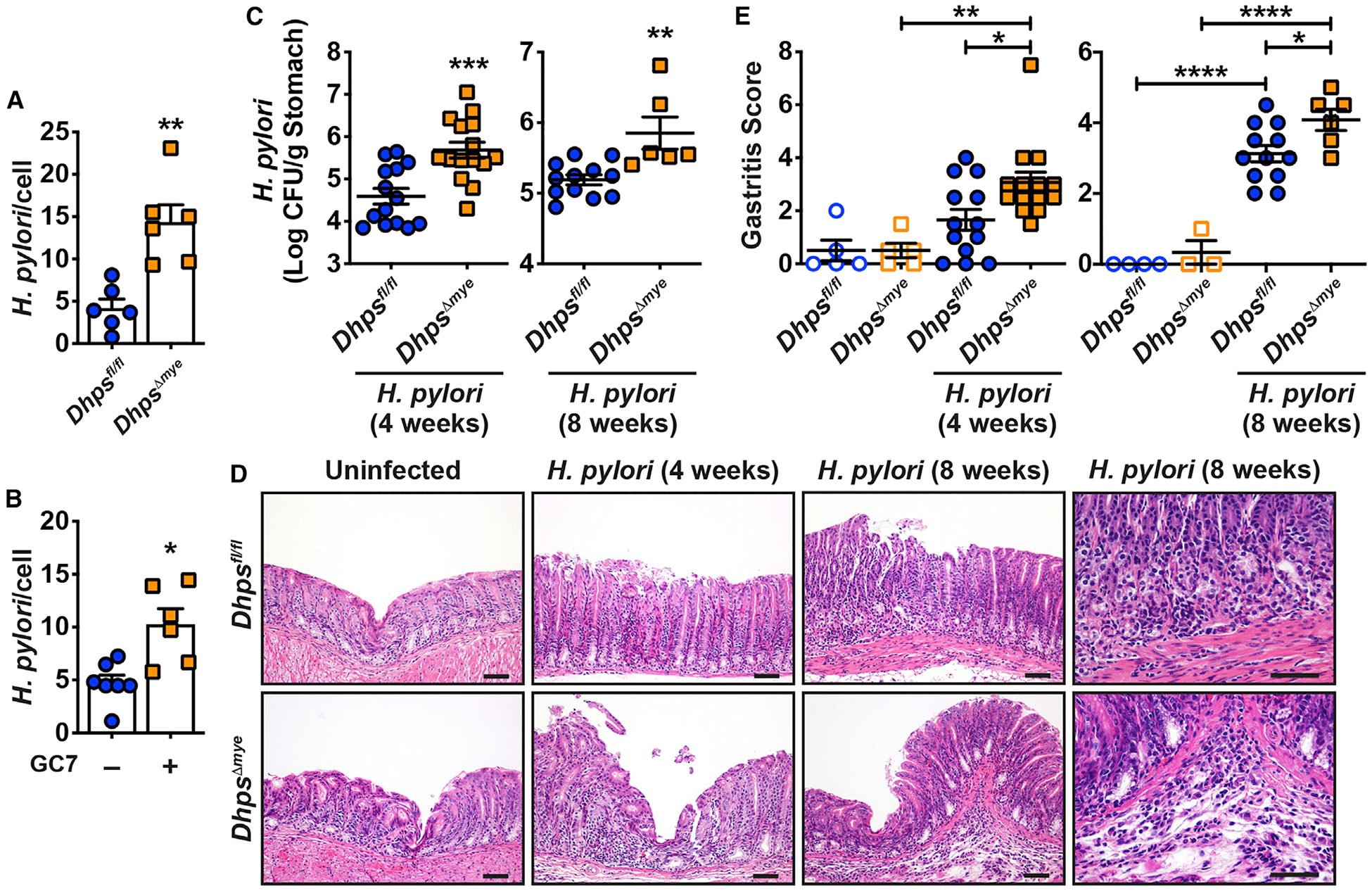
Effect of *Dhps Deletion on Hp* Survival in Macrophage and in the Stomach (A) BMmacs from *Dhps*^*fl/fl*^ and *Dhps*^*Δmye*^ mice were infected with *Hp* at a multiplicity of infection of 100 for 24 h. Cocultures were washed and treated with gentamicin for 1 h. Cells were then harvested, counted, and lysed. The number of live bacteria was determined by counting colony-forming units (CFUs) after plating serial dilutions of cell lysates. Each dot represents BMmacs from one mouse. (B) The same experiments were performed using RAW 264.7 macrophages ± GC7; each dot represents an individual well. (C–E) *Dhps*^*fl/fl*^ and *Dhps*^*Δmye*^ mice were infected or not with *Hp* PMSS1 for 4 and 8 weeks. Gastric colonization was determined by culture (C). H&E staining of the stomach (D) was used to generate the inflammation score of the gastric tissues (E). In all the panels, the mean ± SEM is shown; *p < 0.05, **p < 0.01, ***p < 0.001, and ****p < 0.0001. Scale bars in (D), 50 μm. See also Figures S9 and S10 and Table S7.

Our data indicate that the induction of DHPS in macrophages supports the translation of inducible proteins involved in the defense against pathogens. To verify this postulate, we tested the involvement of hypusination in the antimicrobial effect of macrophages. We observed increased *H. pylori* survival in BMmacs from *Dhps*^*Δmye*^ mice compared with *Dhps*^*fl/fl*^ macrophages (Figure 5A) and in RAW 264.7 cells pre-treated with GC7 compared with untreated macrophages (Figure 5B).

Next, to assess the effect of hypusination in infection *in vivo*, we used a well-established model of *H. pylori* infection in mice (Chaturvedi et al., 2010; Hardbower et al., 2016, 2017). The level of gastric colonization by *H. pylori* at 4 weeks and 8 weeks post-infection was significantly enhanced by 1.1 and 0.8 log, respectively, in animals lacking *Dhps* in myeloid cells compared with *Dhps*^*fl/fl*^ mice (Figure 5C). Concomitantly, hematoxylin and eosin (H&E) images showed more infiltration of immune cells in the gastric mucosa and more gastric hyperplasia in *H. pylori*-infected *Dhps*^*Δmye*^ mice (Figure 5D). Scoring for acute and chronic inflammation in the gastric antrum and corpus (Chaturvedi et al., 2010; Hardbower et al., 2016, 2017) confirmed that gastritis was significantly higher in infected *Dhps*^*Δmye*^ mice than *Dhps*^*fl/fl*^ mice at both time points (Figure 5E). This was confirmed by increased expression of the genes encoding for the chemokines CXCL1 and CXCL2 and for the T cell effectors IL-17 and IL-10 in the gastric mucosa of infected *Dhps*^*Δmye*^ mice (Figure S6A). However, the inducible expression of the genes encoding for NOS2, IL-1β, and interferon (IFN)-γ was not affected by *Dhps* deletion (Figure S6A).

**Figure 6.**
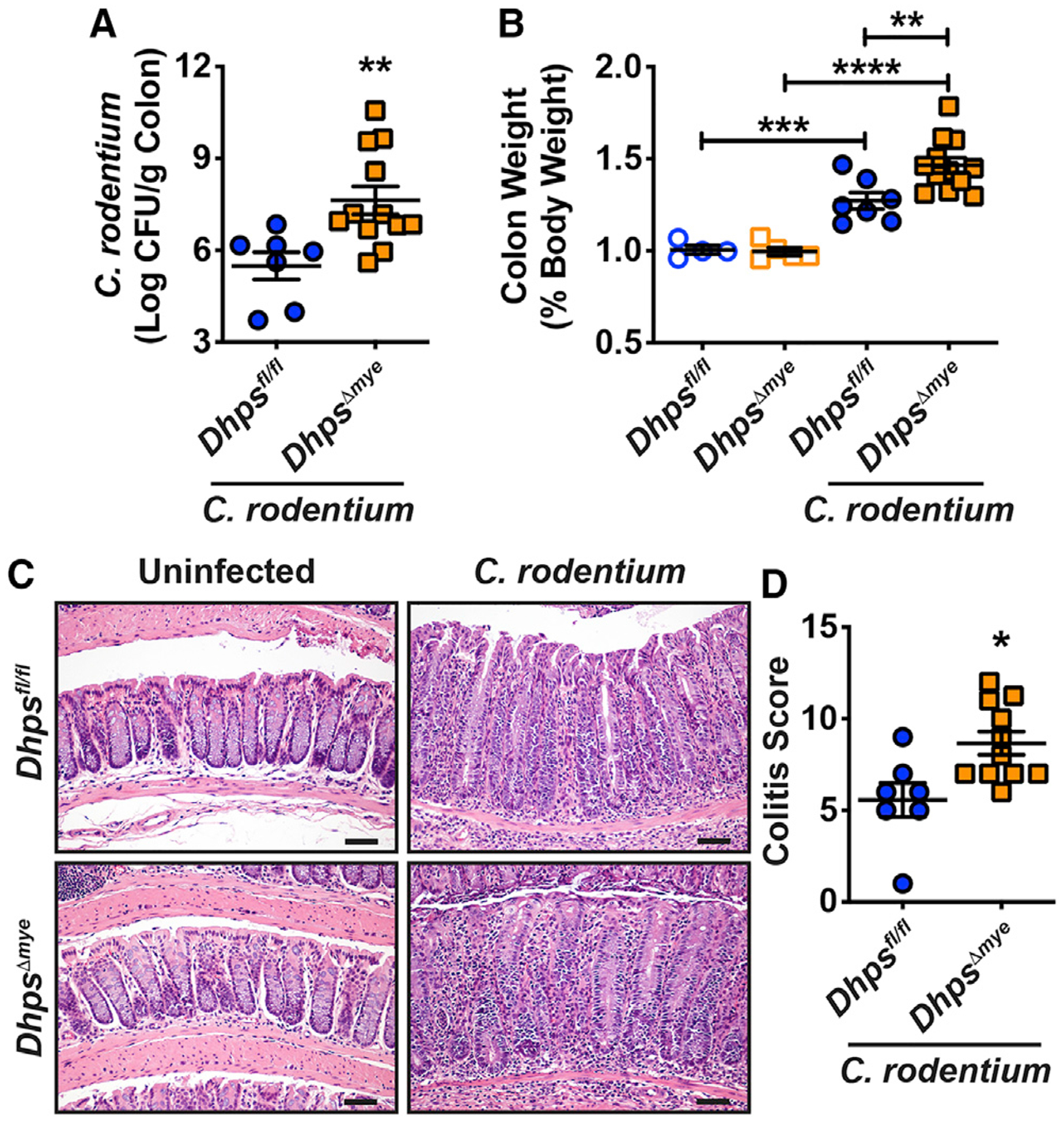
Outcome of *C. rodentium* Infection in *Dhps*^*fl/fl*^ and *Dhps*^*Δmye*^ Mice (A–D) Animals were infected or not with *C. rodentium* for 14 days. Colonization of the colon was assessed by culture (A). Colon was weighed (B). H&E staining of the colon (C) was used to generate the score for colitis (D); the inflammation scores in uninfected *Dhps*^*fl/fl*^ and *Dhps*^*Δmye*^ mice were 0 in stomach and colon. In all the panels, the mean ± SEM is depicted; *p < 0.05, **p < 0.01, ***p < 0.001, and ****p < 0.0001. Scale bars in (C), 50 μm. See also Figure S9.

We then confirmed the role of DHPS in the antibacterial immunity of the gastrointestinal tract using the colonic pathogen *C. rodentium*. Similar to the result with *H. pylori* in the stomach, mice with specific deletion of *Dhps* in myeloid cells exhibited increased colonization by *C. rodentium* (Figure 6A). There was also a significant increase in colon weight (Figure 6B) and histologic colitis in *Dhps*^*Δmye*^ mice (Figures 6C and 6D). *Cxcl1*, *Il1b*, *Ifng*, and *Il17a* mRNA levels were more abundant in the colon of *C. rodentium*-infected *Dhps*^*Δmye*^ mice compared with *Dhps*^*fl/fl*^ mice (Figure S6B).

**Figure 7.**
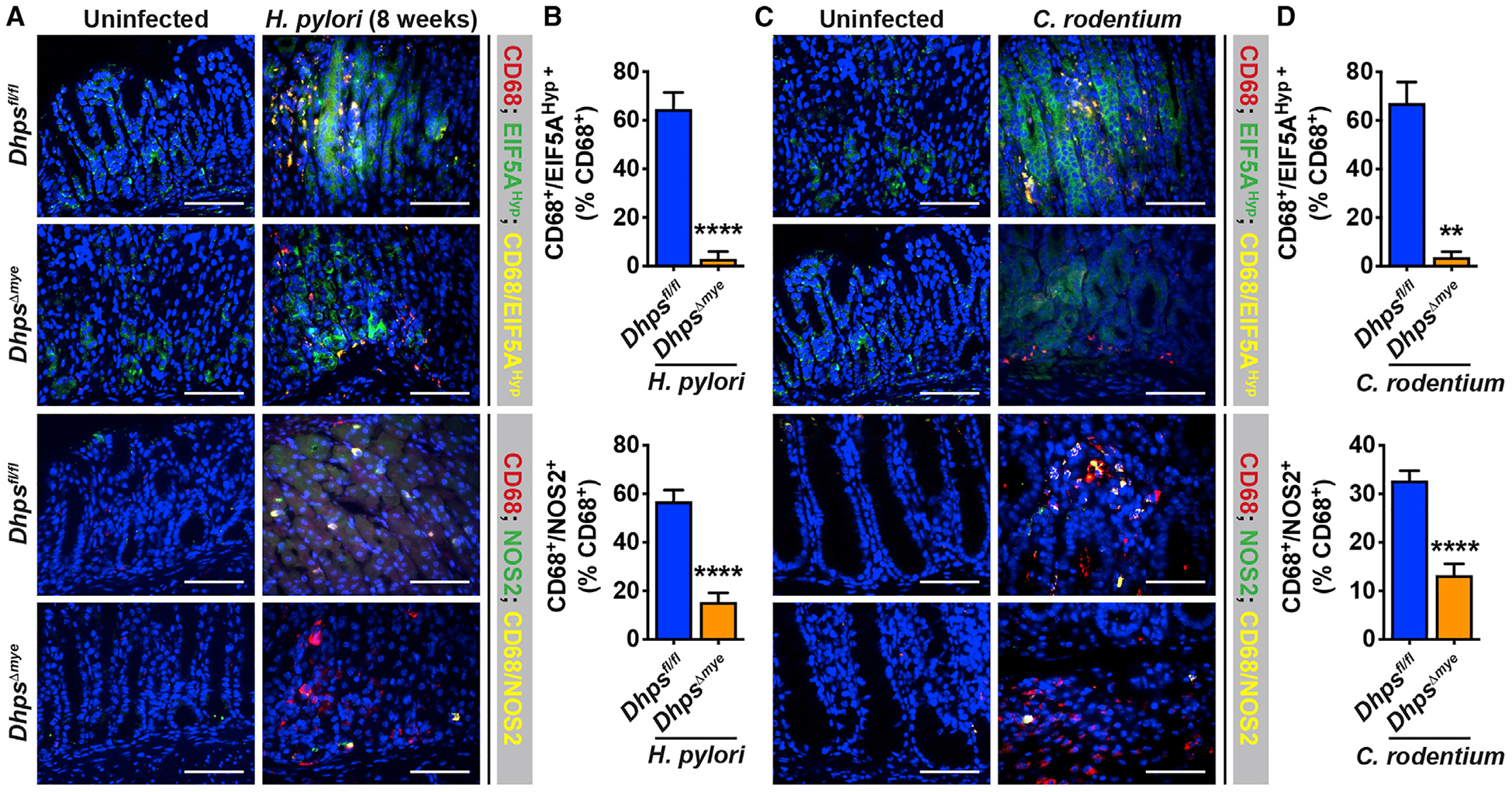
Expression of Immune Effectors Regulated by Hypusination in Infected Tissues (A–D) The gastric tissues of *Dhps*^*fl/fl*^ and *Dhps*^*Δmye*^ mice infected for 8 weeks with *Hp* PMSS1 (A) or the colon of these animals infected with *C. rodentium* for 14 days (C) were immunostained for CD68 and EIF5A^Hyp^, as well as CD68 and NOS2. Scale bars, 50 μm. Quantification of CD68^+^/EIF5A^Hyp +^ cells and CD68^+^/NOS2^+^ cells among the total number of CD68^+^ cells is shown in (B) and (D). The immunofluorescence images are representative of staining performed on two uninfected animals and five infected mice in each group, and the same number of animals was used for quantification. Bars represent mean ± SEM in all graphs; **p < 0.01 and ****p < 0.0001.

Moreover, both *H. pylori* and *C. rodentium* infection of *Dhps*^*fl/fl*^ and *Dhps*^*Δmye*^ mice led to the recruitment of macrophages in the gastric and colonic mucosa, respectively, compared with uninfected animals, as shown by immunofluorescence (IF) for the macrophage marker CD68 (Figures 7A and 7C). We did not observe noteworthy differences in the number of CD68^+^ cells between genotypes (Figures 7A and 7C). Remarkably, macrophages in *H. pylori*- and *C. rodentium*-infected *Dhps*^*fl/fl*^ mice exhibited high levels of EIF5A^Hyp^ (Figures 7A and 7C). By contrast, the level of EIF5A^Hyp^ was strikingly reduced in gastric and colonic CD68^+^ cells from *Dhps*^*Δmye*^ mice infected with *H. pylori* (Figures 7A and 7B) or with *C. rodentium* (Figures 7C and 7D). Note that EIF5A^Hyp^ was also present in other cells from the gastric mucosa of *H. pylori*-infected mice and from the colon of animals infected with *C. rodentium*, but these levels were not altered by myeloid cell *Dhps* deletion (Figure 7C). Of interest, the levels of NOS2, which have been found to be regulated by hypusination, were also increased in CD68^+^ macrophages in *H. pylori*- and *C. rodentium*-infected *Dhps*^*fl/fl*^ mice and were markedly reduced in *Dhps*^*Δmye*^ mice (Figures 7A–7D).

Lastly, we assessed the effect of *Dhps* deletion in macrophages on the gastric metabolomic signatures. There were 267 positively charged and 341 negatively charged metabolites significantly affected in infected *Dhps*^*Δmye*^ mice compared with *Dhps*^*fl/fl*^ mice (Figure S7). As an example, the level of spermidine, the substrate for DHPS, was increased in the stomach of *Dhps*^*Δmye*^ mice. Conversely, the NOS2 product citrulline, and the oxidized from of glutathione, which is a reliable indicator of oxidative stress, were less abundant in the *Dhps*^*Δmye*^ animals (Figure S7). The analysis of the metabolomic pathways (Table S7) evidenced that the citrulline-NO cycle was significantly affected by infection in *Dhps*^*fl/fl*^ mice, but not in *Dhps*^*Δmye*^ mice. We also found that the TCA cycle was affected by *Dhps* deletion in myeloid cells (Table S7).

## DISCUSSION

Hypusination of EIF5A is a unique mechanism that controls the translation of specific proteins. In this study, we have utilized an unbiased approach using specific genetic deletion of *Dhps* and a DHPS inhibitor to probe the role of the spermidine-EIF5A^Hyp^ axis in macrophage responses to pathogenic bacteria. Increased DHPS expression and levels of EIF5A^Hyp^ were observed in macrophages infected *ex vivo* with *H. pylori* and in gastric CD68^+^ cells from *H. pylori*- and *C. rodentium*-infected mice. This results in the translation of immune effectors principally involved in microbial clearance and cell death, as underlined by the IPA of the proteomic data. Importantly, these results were obtained with the human gastric pathogen *H. pylori* and the murine colon bacterial pathogen *C. rodentium*, providing strong evidence that our finding is not limited to one pathogen but concerns the global innate response of macrophages. Lastly, our data showing increased colonization of *Dhps*^*Δmye*^ mice by both pathogens endorse the *in vivo* relevance of the paramount role of hypusination in the first line of defense of macrophages against bacterial infection of the gastrointestinal mucosa. Our present findings emphasize one more step in the complex regulation of the immune response by polyamines: first, putrescine regulates the transcription of innate effectors (Hardbower et al., 2017); and second, spermidine controls translation through hypusination, as shown herein. Of importance, we found that hypusination is reduced in infected BMmacs from *Odc*^*Δmye*^ mice, but not completely suppressed, consistent with the partial reduction of spermidine concentration by *Odc* deletion in macrophages (Hardbower et al., 2017). This indicates that ODC-derived putrescine affects transcription, but does not sufficiently alter translation by modulation of hypusination. Taken together, our data herein and our prior study (Hardbower et al., 2017) illustrate a complex immunologic rheostat for macrophage innate immune effectors, where putrescine from ODC regulates transcription, and spermidine, acting through DHPS/hypusination, regulates translation, providing robust biological control.

Previous reports have evidenced that EIF5A knockdown using small hairpin RNA in cervical cancer cells (Mémin et al., 2014), pancreatic ductal adenocarcinoma cells (Fujimura et al., 2015), and HeLa cells (Mandal et al., 2016) regulates proteins involved in migration, invasion, proliferation, endoplasmic reticulum (ER) stress, and/or unfolded protein response. Moreover, mitochondrial enzymes and proteins involved in the TCA cycle are affected by GC7 in M0 and M2 macrophages and by *Eif5a* and *Dhps* silencing in murine embryonic fibroblasts (Puleston et al., 2019). We have identified a series of bacteria-induced proteins that are regulated by DHPS function in macrophages.

Interestingly, it has also been shown that hypusination of EIF5A is enhanced in alternatively activated macrophages, in which the arginase-ODC metabolic pathway is strongly activated, but not during the classical macrophage activation with lipopolysaccharide (LPS)/IFN-γ (Puleston et al., 2019). However, in the context of infected macrophages, we propose now that DHPS induction supports increased hypusination of EIF5A and translation of antimicrobial factors. Note that in this prior study (Puleston et al., 2019), the authors did not identify the proteins involved in the antimicrobial response since they were not induced by M2 stimulation. In this previous work, the authors demonstrated that the TCA cycle was dampened in M0, M1, and M2 macrophages treated with GC7 (Puleston et al., 2019). Accordingly, our proteomic analysis of RAW 264.7 cells ± GC7 evidenced that the enzymes SDHB, SUCB1, SUCB2, ODPA, and ODPB, which all belong to the TCA cycle, are downregulated by GC7 in unstimulated cells (Table S4). However, since these enzymes were also reduced by *H. pylori* infection, we suggest that the control of infection by hypusination is not dependent on the TCA cycle.

In our study, numerous immune effectors regulated by hypusination are involved in the host defense against pathogens. Notably, the protein levels of NOS2, ASSY, and IRG1 were markedly reduced during *H. pylori* infection in *Dhps*^*Δmye*^ macrophages compared with *Dhps*^*fl/fl*^ and in GC7-treated macrophages. NOS2 expression is a hallmark of macrophage activation by pathogen-associated molecular patterns and proinflammatory cytokines (MacMicking et al., 1997) and has been already reported to be regulated by hypusination in pancreatic β cells (Maier et al., 2010) and in murine macrophages stimulated with the mycobacterial cell wall component trehalose-6,6′-dimycolate (Lee et al., 2016). NOS2-derived high-output NO production has been implicated in the killing of various parasites and bacteria (Duleu et al., 2004; El Kasmi et al., 2008; Qualls et al., 2012), including *H. pylori* (Gobert et al., 2001). Similarly, ASSY converts citrulline into arginine, the NOS2 substrate, to increase the NO-dependent killing of bacteria (Qualls et al., 2012). In addition, the innate effector IRG1 has been shown to be induced in RAW 264.7 cells and primary macrophages stimulated by LPS and IFN-γ (Strelko et al., 2011), tumor necrosis factor alpha, Toll-like receptor (TLR) 2, or TLR4 signaling (Degrandi et al., 2009) and leads to the synthesis of itaconic acid (Basler et al., 2006; Degrandi et al., 2009; Strelko et al., 2011). This metabolite inhibits isocitrate lyase, a key enzyme that is necessary for bacterial growth (Michelucci et al., 2013). Interestingly, it has been recently shown that the CRISPR-Cas9 deletion of both NOS2 and IRG1 reduced the killing of *Legionella pneumophila* by murine BMmacs stimulated by IFN-γ (Price et al., 2019). In addition, the maximal killing of *L. pneumophila* by macrophages required the expression of four other immune effectors: CASP11, NOX2, IRGM3, and IRGM1 (Price et al., 2019); this last protein was also found to be regulated by hypusination in our experiments. Thus, our data highlight that hypusination is a major regulator of host defense by affecting the translation of multiple antimicrobial effectors in macrophages; this postulate was supported by our finding that bacterial survival is increased in macrophages with DHPS knockout or inhibition. Of note, we also observed that hypusination regulates mediators involved in antiviral response, including the receptor of viral nucleic acids IFIH1, the antiviral exoribonuclease ISG20, the GTPases GBP2 and GBP4, and the antiviral proteins RRSAD2, IFIT2, and IFIT3, further indicating that hypusination is a general control mechanism of the overall innate response of macrophages.

The abundance of the proteins IIGP1, IRGM1, CLEC4E, and SQSTM, which are all implicated in autophagy, was affected by DHPS deletion or inhibition in infected macrophages, and we also found that GC7 reduced *H. pylori*-mediated autophagy. Remarkably, these effectors also play a fundamental role in the destruction of microorganisms (Al-Zeer et al., 2009; MacMicking et al., 2003; Pahari et al., 2019; Watson et al., 2012). Intriguingly, spermidine, the DHPS substrate, supports autophagy in various cells including human peripheral blood mononuclear cells (Eisenberg et al., 2009) or in a human colon cancer cell line (Morselli et al., 2011). In the same way, mice treated with spermidine exhibit prolonged lifespan and improved cardiomyocyte function due to increased cardiac autophagic flux (Eisenberg et al., 2016). Furthermore, it has been recently observed that hypusinated EIF5A controls the synthesis of the transcription factor EB, a master regulator of genes encoding mediators of autophagy, in naive B cells (Zhang and Simon, 2020). This transcription factor was not evidenced to be regulated by infection and/or inhibition of DHPS in our study, suggesting that EIF5A^Hyp^ may regulate autophagy by various pathways. Nonetheless, we contend that hypusination is also involved in the autophagy-dependent clearance of pathogenic bacteria by macrophages.

Polyamine metabolism plays a fundamental role in the outcome of bacterial infections, including those of the gastrointestinal tract (Chaturvedi et al., 2010; Gobert et al., 2018; Hardbower et al., 2017). This report highlights that the spermidine-hypusine metabolic pathway represents an unrecognized foundation of the antimicrobial armature. It is important to underline that reduced levels of EIF5A^Hyp^ in tissue macrophages led not only to increased colonization by *H. pylori* and *C. rodentium* but also to enhanced inflammation. We therefore propose that the loss of innate host defense in *Dhps*^*Δmye*^ macrophages led to the uncontrolled development of infection, which results in increased inflammation. Epithelial responses and chronic inflammation are also critical for the outcome of gastrointestinal infections. Hypusination has been shown to support the development of cancerous cells (Nakanishi and Cleveland, 2016); in contrast, it has also been recently evidenced that the polyamine-EIF5A^Hyp^ metabolic pathway exhibits tumor suppressor functions in a mouse lymphoma model (Scuoppo et al., 2012). Given that *H. pylori* is the main causative agent for the development of gastric cancer (Parsonnet et al., 1991), the relative functional contribution of hypusination in epithelial, myeloid, and T cells in host defense against carcinogenetic events deserves further investigation and is underway in our laboratory.

## STAR★METHODS

### RESOURCE AVAILABILITY

#### Lead contact

Further information and requests for resources and reagents should be directed to and will be fulfilled by the Lead Contact, Keith T. Wilson (keith.wilson@vumc.org).

#### Materials availability

*Dhps*^*fl/fl*^;*Lyz2*^*cre/+*^ and *Dhps*^*fl/fl*^;*Lyz2*^*cre/cre*^ mice were generated for this study and are available upon request.

#### Data and code availability

The mass spectrometry proteomics data regarding BMmacs (Tables S1 and S2) and RAW 264.7 cells (Table S4) have been deposited to the ProteomeXchange Consortium via the PRIDE partner repository (Perez-Riverol et al., 2019), and the accession numbers are ProteomeXchange Consortium: PXD018187 and ProteomeXchange Consortium: PXD010082, respectively. The accession number for the metabolomics data reported in this paper is EMBL-EBI MetaboLights: MTBLS607.

### EXPERIMENTAL MODEL AND SUBJECT DETAILS

#### Human tissues

Biopsies from gastric tissues were obtained from human subjects in Colombia as described (Mera et al., 2018), under protocols approved by the ethics committees of the local hospitals and of the Universidad del Valle in Cali, Colombia, as well as the Institutional Review Board at Vanderbilt University. We used biopsies from 5 uninfected subjects (4 females and 1 male) aged 26–64 years (mean ± standard deviation: 47.2 ± 13.6 years) and 9 *H. pylori*-infected patients (5 females and 4 males) aged 26–45 years (mean ± standard deviation: 34.6 ± 6.1 years).

#### Animals

Mice were used under protocols M/05/176 and M/08/124 approved by the Institutional Animal Care and Use Committee at Vanderbilt University and Institutional Biosafety Committee, and the Research and Development Committee of the Veterans Affairs Tennessee Valley Healthcare System. Procedures were performed in accordance with institutional policies, AAALAC guidelines, the AVMA Guidelines on Euthanasia, NIH regulations (Guide for the Care and Use of Laboratory Animals), and the United States Animal Welfare Act (1966). To achieve specific deletion of *Dhps* in myeloid cells, we crossed C57BL/6J *Dhps*^*fl/fl*^ mice, in which exons 2 to 7 of the *Dhps* gene were flanked by Cre recombinase recognition sequences (Levasseur et al., 2019), to mice with the myeloid driver *Lyz2*^*cre*^. *Dhps*^*fl/fl*^;*Lyz2*^*cre/+*^ mice were then crossed to each other to generate littermate *Dhps*^*fl/fl*^;*Lyz2*^*cre/cre*^, *Dhps*^*fl/fl*^;*Lyz2*^*cre/+*^ and *Dhps*^*fl/fl*^ mice in expected proportions (25%, 52% and 23%, respectively). Thereafter, we used littermates, namely *Dhps*^*fl/fl*^ mice as controls, *Dhps*^*fl/fl*^;*Lyz2*^*cre/+*^ mice, and *Dhps*^*fl/fl*^;*Lyz2*^*cre/cre*^ mice, which we termed *Dhps*^*Δmye*^ mice. We also used *Odc*^*fl/fl*^ and *Odc*^*Δmye*^ mice that are bred in our animal facility (Hardbower et al., 2017). Adult mice (8–12 weeks) of both sexes were used for isolation of BMmacs. Only male mice (8–12 weeks) were used for infection with *H. pylori* and *C. rodentium*.

#### Isolation of myeloid cells

BMmacs and BMDCs from *Dhps*^*fl/fl*^, *Dhps*^*fl/fl*^;*Lyz2*^*cre/+*^, and *Dhps*^*fl/fl*^;*Lyz2*^*cre/cre*^ mice were isolated and differentiated as described (Hardbower et al., 2016).

#### Bacteria

The *cagA*^+^ *H. pylori* strains PMSS1 (Arnold et al., 2011) and *C. rodentium* DBS100 (Barthold et al., 1976) were grown on Tryptic Soy agar plates containing 10% sheep blood and MacConkey agar plates, respectively. Bacteria were harvested directly from plates to infect the cells. From the plates, *H. pylori* and *C. rodentium* were also grown in Brucella broth containing 10% FBS and in Luria-Bertani broth liquid medium overnight, respectively; they were then diluted to an *A*_600__nm_ of 0.1 in these media, grown, and harvested at the exponential phase to infect mice.

### METHOD DETAILS

#### Infections

C57BL/6J, *Dhps*^*fl/fl*^, *Dhps*^*fl/fl*^;*Lyz2*^*cre/+*^, *Dhps*^*fl/fl*^;*Lyz2*^*cre/cre*^, *Odc*^*fl/fl*^, and *Odc*^*Δmye*^ mice were bred in our animal facility. Mice were fed *ad libitum* with regular 5L0D chow (LabDiet). For infections, male mice at 8–12 weeks of age were randomly distributed in the different groups and infected by oral gavage with *i*) 10^9^ *H. pylori* PMSS1 in 0.2 mL Brucella broth, two times every 2 days (Sierra et al., 2018), or *ii*) 5×10^8^ *C. rodentium* DBS100 in in 0.2 mL Luria-Bertani broth (Gobert et al., 2018; Hardbower et al., 2016). Control mice were gavaged with the corresponding broth alone. In both models, mice were monitored daily and those that showed extreme distress, became moribund, or lost more than 20% of initial body weight were euthanized. Animals were sacrificed after 4 or 8 weeks (*H. pylori*) and 14 days (*C. rodentium*). Stomachs and colons were harvested and analyzed as described (Chaturvedi et al., 2010; Gobert et al., 2018; Hardbower et al., 2016, 2017). The number of bacteria in gastric and colon tissues was determined by counting the CFUs after plating serial dilutions of homogenized tissues.

#### Histopathology

Histologic assessments were performed by a gastrointestinal pathologist (M.B.P.) in a blinded manner. At necropsy, stomach and colon segments were fixed in 10% neutral buffered formalin and stained with H&E. For *H. pylori*-infected mice, inflammation was assessed on a longitudinal strip using the following histopathological features: acute and chronic inflammation of the antrum and the corpus regions of the stomach (0–3 for each), leading to a final 0–12 score (Chaturvedi et al., 2010; Hardbower et al., 2016, 2017). For *C. rodentium* infection, the histologic colitis score (0–18) was the sum of acute and chronic inflammation scores (0–3 for each) multiplied by extent of inflammation (0–3), as reported (Chaturvedi et al., 2010; Gobert et al., 2018; Hardbower et al., 2016, 2017).

#### Immunostaining

Immunofluorescent staining for EIF5A^Hyp^, NOS2, and the macrophage marker CD68 was performed on murine and human gastric tissues as described (Hardbower et al., 2016, 2017) using the following antibodies (see the Key Resources Table): Rabbit polyclonal anti-EIF5A^Hyp^, 1/2000; rabbit polyclonal anti-NOS2, 1/1000; rabbit polyclonal anti-mouse CD68, 1/100; mouse monoclonal anti-Human CD68, ready to use; donkey anti-mouse IgG, Alexa fluor 555-labeled, 1/500; goat anti-rabbit IgG, Alexa fluor 488-labeled, 1/400; goat anti-rabbit IgG, Alexa fluor 555-labeled, 1/500. Quantification was performed by two blinded observers and the scoring was averaged.

#### Co-culture of myeloid cells and bacteria

We used BMmacs, BMDCs, and the murine macrophage cell line RAW 264.7 that was maintained in DMEM containing 10% FBS, 10 mM HEPES and 1 mM sodium pyruvate. Cells were stimulated with *H. pylori* at a multiplicity of infection of 10–100 for 6 or 24 h. For *C. rodentium*, macrophages were infected for 3 h with a multiplicity of infection of 1–10; then, cocultures were washed and a fresh media containing gentamycin, penicillin, and streptomycin was added to each well for 3 or 21 more h. In some experiments, cells were pre-treated with GC7 (Biosearch Technologies; 10 μM), Bay 11–7082 (Calbiochem; 5 μM), ERK inhibitor (Calbiochem; 20 μM), LY294002 (Calbiochem; 10 μM), PD98059 (Calbiochem; 10 μM), or SB203580 (Calbiochem; 2 μM) 1 h before infection. RAW264.7 cells tested negative for mycoplasma contamination using the Lookout Mycoplasma detection kit (Sigma-Aldrich).

To determine the survival of bacteria in macrophages, cocultures were washed thoroughly with PBS after 24 h, incubated 1 h with 200 μg/ml gentamicin, and lysed in 0.1% saponin for 30 min at 37°C (Gobert et al., 2014). The number of bacteria in each lysate was determined by counting the CFUs after plating serial dilutions on blood agar plates.

#### Analysis of mRNA levels

Total RNA was isolated using the RNeasy Mini kit (QIAGEN). Reverse transcription was performed using Superscript II Reverse Transcriptase and Oligo dT (Invitrogen). mRNAs were amplified by real-time PCR using the iQ^™^ SYBR Green kit (Bio-Rad) (Gobert et al., 2014) and the primers listed in Key Resources Table.

#### Proteomics and IPA

Proteomic analysis was performed as described (Gobert et al., 2019; Noto et al., 2019). After infections, macrophages were lysed in 50 mM Tris-HCl pH 7.6, 150 mM NaCl, 1% NP-40, and 2 mM EDTA and protein concentrations were determined by BCA. Protein samples were precipitated with ice-cold acetone overnight at −20°C. Precipitates were washed with cold acetone, dried, and reconstituted in 8 M urea in 250 mM TEAB (pH 8.0). Samples were reduced with 50 mM TCEP, alkylated with 200 mM MMTS, and diluted with TEAB to obtain a final solution containing 2 M urea, and digested with sequencing-grade trypsin overnight. Quantitative proteomics analysis was performed using iTRAQ. For 25 μg of protein, 1 unit of 4-plex iTRAQ labeling reagent (SCIEX) was used. Labeling reagent was reconstituted in ethanol such that each protein sample was labeled at a final concentration of 90% ethanol, and labeling was performed for 2 h. Four-plex iTRAQ experiments were conducted. The resulting labeled peptides were then desalted by a modified Stage-tip method. Following desalting, peptides were reconstituted in 0.1% formic acid, and peptides were loaded onto a self-packed biphasic C18/SCX MudPIT column using a helium-pressurized cell. Using a Dionex Ultimate 3000 nanoLC and autosampler, MudPIT analysis was performed with a 15-step salt pulse gradient. Following each salt pulse delivered by the autosampler, peptides were gradient-eluted from the reverse analytical column at a flow rate of 350 nl/min. Mobile phase solvents consisted of 0.1% formic acid, 99.9% water (solvent A) and 0.1% formic acid, 99.9% acetonitrile (solvent B). For the peptides from the first 13 SCX fractions, the reverse phase gradient consisted of 2%–50% B in 83 min, followed by a 10 min equilibration at 2% B. For the last 2 SCX-eluted peptide fractions, the peptides were eluted from the reverse phase analytical column using a gradient of 2%–98%B in 83 min, followed by a 10 min equilibration at 2% B. A Q Exactive Plus or HF mass spectrometer (Thermo Scientific), equipped with a nanoelectrospray ionization source, was used to mass analyze the eluting peptides. The Q Exactive instrument was operated in data-dependent mode acquiring HCD MS/MS after each MS1 scan on the 15 most abundant ions using an MS2 target of 10^5^ ions. The HCD-normalized collision energy was set to 30, dynamic exclusion was set to 30 s, and peptide match and isotope exclusion were enabled.

Peptide/protein identifications and quantitative analysis were performed using Spectrum Mill (Agilent). MS/MS spectra were searched against a subset of the UniProt KB protein database containing *Mus musculus* and *Helicobacter pylori* 26695 proteins. Autovalidation procedures in Spectrum Mill were used to filter the data to < 1% false discovery rates at the protein and peptide level. Log2 protein ratios were fit to a normal distribution using non-linear (least-squares) regression. The calculated mean derived from the Gaussian fit was used to normalize individual log2 ratios for each quantified protein. The normalized log2 ratios were then fit to a normal distribution, and the mean and standard deviation values derived from the Gaussian fit of the normalized ratios were used to calculate p values. Subsequently, p values were corrected for multiple comparisons by the Benjamini-Hochberg method.

IPA software (QIAGEN) was used for the functional interpretation of differential expression results obtained from the aforementioned proteomic analyses. The pathways related to Diseases and Functions and the protein interaction networks were generated. The statistical significance for each assignment was expressed by a corresponding p values calculated using Fisher’s exact test.

#### Untargeted Metabolomics

Gastric tissues were homogenized by sonication in water:methanol (9:1) containing 50 mM ammonium acetate (pH~6) to yield a tissue density of 50 mg/ml. A portion of the homogenate (100 μL) was combined with HPLC-grade methanol (300 μL), vortexed vigorously, and centrifuged at 10,000 × *g*. An aliquot of the supernatant (100 μL) was diluted with an equal volume of HPLC-grade acetonitrile. Discovery metabolomics data were acquired using a Vanquish ultrahigh performance liquid chromatography (UHPLC) system interfaced to a Q Exactive HF quadrupole/orbitrap mass spectrometer (Thermo Fisher Scientific). Each sample was injected twice. The first injection was made in positive ESI mode; the second injection was made in negative mode. A Zic-cHILIC analytical column (3 μm, 2.1 × 150 mm; Merck SeQuant) was used for all chromatographic separations. Mobile phases were made up of 0.2% acetic acid and 15 mM ammonium acetate in (A) H_2_O:CH_3_CN (9:1) and in (B) CH_3_CN:CH_3_OH:H_2_O (90:5:5). The flow rate was maintained at 300 μl/min, the total chromatographic run time was 20 min, and the sample injection volume was 10 μl. Mass spectra were acquired over a precursor ion scan range of m/z 100 to 1,200 at a resolving power of 30,000 using the following ESI source parameters: spray voltage 5 kV (3 kV in negative mode); capillary temperature 300°C; S-lens RF level 60 V; N2 sheath gas 40; N2 auxiliary gas 10; auxiliary gas temperature 100°C. MS/MS spectra were acquired for the top-five most abundant precursor ions with an MS/MS AGC target of 10^5^, a maximum MS/MS injection time of 100 ms, and a normalized collision energy of 30. Chromatographic alignment, peak picking, and statistical comparisons were performed using XCMS (https://xcmsonline.scripps.edu).

#### Western blot analysis

Macrophages were lysed using RIPA buffer containing the Protease Inhibitor Cocktail (Set III, Calbiochem) and the Phosphatase Inhibitor Cocktail (Set I, Calbiochem). Protein concentrations were determined using the BCA Protein Assay (Pierce). Western blotting was performed using 2 μg of protein per lane for all the blots, except for the EIF5A and EIF5A^Hyp^ blots for which 10 μg was used. The concentration of the primary and secondary Abs (see the Key Resources Table) were as follows: Rabbit polyclonal anti-DHPS, 1/2000; rabbit polyclonal anti-EIF5A^Hyp^, 1/10000; rabbit monoclonal anti-EIF5A, 1/2000; rabbit polyclonal anti-NOS2, 1/2000; rabbit monoclonal anti-IRG1, 1/2000; rabbit polyclonal anti-IRGM1, 1/2000; rabbit polyclonal anti-SQSTM, 1/10000; mouse monoclonal anti-β-actin, 1/20000; goat anti-rabbit IgG, HRP-labeled, 1/5000; goat anti-mouse IgG, HRP-labeled, 1/5000. Densitometric analysis of western blots was performed with ImageJ 1.53a software.

#### NOS2 translation assay

BMmacs from *Dhps*^*fl/fl*^ and *Dhp*^*Δmye*^ mice were infected or not with *H. pylori* for 18 h. Cells were then washed with PBS and methionine-free, cysteine-free DMEM (GIBCO) was added to the cells for 2 h. This medium was then replaced by methionine-free, cysteine-free DMEM supplemented with 50 μM Click-IT homopropargylglycine (Invitrogen) for 4 h. Proteins were extracted using 1% SDS in 50 mM Tris-HCl containing the Protease Inhibitor Cocktail (Set III, Calbiochem) and the Phosphatase Inhibitor Cocktail (Set I, Calbiochem). Proteins (50 μg) were labeled with biotin azide (Invitrogen) using the Click Chemistry Reagents (Invitrogen), precipitated by methanol, resuspended in 50 mM Tris-HCl, and immunoprecipitated using a NOS2 Ab (Millipore) and the PureProteome Protein A Magnetic Bead System (Millipore). The resulting precipitates were used for western blotting using 4plus Streptavidin HRP Label (Biocare Medical).

#### Determination of autophagy

We determined autophagy in RAW 264.7 cells infected or not with *H. pylori*, in the presence or absence of GC7, by flow cytometry using the CYTO-ID® Autophagy Detection Kit (Enzo).

#### Measurement of NO_2_^−^

NO_2_^−^ concentration was determined by the standard Griess assay (Promega)

### QUANTIFICATION AND STATISTICAL ANALYSIS

Statistical analyses were performed with GraphPad Prism 8.4.3 and the significance level was set as p < 0.05. All the data shown represent the mean ± SEM. Data that were not normally distributed according to the D’Agostino & Pearson normality test were log or square root transformed. Student’s t test or ANOVA with the Tukey test were used to determine significant differences between two groups or to analyze significant differences among multiple test groups, respectively. All statistical tests were two-sided.

## Supplementary Material

1

2

3

4

5

6

7

8

## Figures and Tables

**Table:** KEY RESOURCES TABLE

REAGENT or RESOURCE	SOURCE	IDENTIFIER
Antibodies		
Rabbit polyclonal anti-EIF5A^Hyp^ antibody	Nishiki et al., 2013	N/A
Rabbit polyclonal anti-EIF5A antibody	Abcam	Cat#ab227537
Rabbit polyclonal anti-DHPS	Abcam	Cat#ab202133
Rabbit polyclonal anti-NOS2	Millipore	Cat#ABN26; RRID: AB_10805939
Rabbit monoclonal anti-IRG1	Abcam	Cat#ab222411; RRID: AB_2868510
Rabbit polyclonal anti-IRGM1	Abcam	Cat# ab118569; RRID: AB_10901765
Rabbit polyclonal anti-SQSTM	Proteintech	Cat#55274–1-AP; RRID: AB_11182278
Rabbit polyclonal anti-mouse CD68	Boster Biological	Cat#A1518
Mouse monoclonal anti-human CD68	Biocare Medical	Cat#PM033AA
Mouse monoclonal anti-β-actin	Sigma	Cat#A5316
Goat anti-rabbit IgG, HRP-labeled	Jackson ImmunoResearch	Cat#111-035-003; RRID: AB_2313567
Goat anti-mouse IgG, HRP-labeled	Jackson ImmunoResearch	Cat#115-035-003; RRID: AB_10015289
Donkey anti-mouse IgG, Alexa fluor 555-labeled	Life Technologies	Cat#A-31570; RRID: AB_2536180
Goat anti-rabbit IgG, Alexa fluor 488-labeled	Life Technologies	Cat#A-11008; RRID: AB_143165
Goat anti-rabbit IgG, Alexa fluor 555-labeled	Life Technologies	Cat#A-21429; RRID: AB_2535850
Bacterial and Virus Strains		
*Helicobacter pylori* strain PMSS1	Arnold et al., 2011	N/A
*Citrobacter rodentium* strain DBS100	Barthold et al., 1976	N/A
Biological Samples		
Human gastric biopsies	Universidad del Valle, Cali, Colombia	N/A
Chemicals, Peptides, and Recombinant Proteins		
Bay 11–7082	Calbiochem	Cat#196870
ERKi	Calbiochem	Cat#3328006
LY294002	Calbiochem	Cat#440206
PD98059	Calbiochem	Cat#513000
SB203580	Calbiochem	Cat#559395
GC7	Biosearch Technologies	Cat#G-1000
Oligo dT	Invitrogen	Cat#18418020
4plus Streptavidin HRP Label	Biocare Medical	Cat#HP604H
Critical Commercial Assays		
RNeasy Mini kit	QIAGEN	Cat#74106
Superscript II Reverse Transcriptase	Invitrogen	Cat#18064022
iQ^™^ SYBR Green kit	Bio-Rad	Cat#1708880
BCA Protein Assay Kit	Pierce	Cat#23225
Griess Reagent System	Promega	Cat#G2930
Protease Inhibitor Cocktail Set III	Calbiochem	Cat#539134
Phosphatase Inhibitor Cocktail Set I	Calbiochem	Cat#539131
CYTO-ID® Autophagy Detection Kit	Enzo	Cat#ENZ-51031–0050
Click-IT L-Homopropargylglycine	Invitrogen	Cat#C10186
Biotin azide	Invitrogen	Cat#B10184
Click-iT Protein Reaction Buffer Kit	Invitrogen	Cat#C10276
PureProteome Protein A Magnetic Bead System	Millipore	Cat#LSKMAGA02
Lookout Mycoplasma detection kit	Sigma-Aldrich	Cat#D9307
Deposited Data		
Proteomics dataset from BMmacs from *Dhps*^*fl/fl*^ and *Dhps*^*Δmye*^ *± H. pylori* or *C. rodentium*	This paper	ProteomeXchange Consortium (http://www.proteomexchange.org): PXD018187
Proteomics dataset from RAW 264.7 cells ± *H. pylori* ± GC7	This paper	ProteomeXchange Consortium (http://www.proteomexchange.org): PXD010082
Metabolomics dataset	This paper	EMBL-EBI MetaboLights (https://www.ebi.ac.uk/metabolights/): MTBLS607
Experimental Models: Cell Lines		
*Mus musculus* RAW 264.7 cells	ATCC	Cat#TIB-71
Experimental Models: Organisms/Strains		
C57BL/6J *Dhps*^*fl/fl*^	Levasseur et al., 2019	N/A
C57BL/6J *Lyz2*^*cre/cre*^	The Jackson Laboratory	Cat#004781
C57BL/6J *Dhps*^*fl/fl*^*;Lyz2*^*cre/+*^	This paper	N/A
C57BL/6J *Dhps*^*fl/fl*^*;Lyz2*^*cre/cre*^	This paper	N/A
C57BL/6J *Odc*^*fl/fl*^	Hardbower et al., 2017	N/A
C57BL/6J *Odc*^*Δmye*^	Hardbower et al., 2017	N/A
Oligonucleotides		
Murine *Dhps* (F)	This paper	CTTCCAGGCTACCAACTTCG
Murine *Dhps* (R)	This paper	GAGTCAGGTCTGCGTGATGA
Murine *Dohh* (F)	This paper	ACATGTGCAGGACCCTACCT
Murine *Dohh* (R)	This paper	GCACGGTATCGCTCAAAGA
Murine *Nos2* (F)	This paper	CACCTTGGAGTTCACCCAGT
Murine *Nos2* (R)	This paper	ACCACTCGTACTTGGGATGC
Murine *Irg1* (F)	This paper	ATCTTGGACCTGGGGTCAG
Murine *Irg1* (R)	This paper	TAAAGGCCACATCCTGCTG
Murine *Irgm1* (F)	This paper	GACTCTGGCAATGGCATGT
Murine *Irgm1* (R)	This paper	ACAGCACCACATTGGGAAA
Murine *Sqstm1* (F)	This paper	aagaacgcgtgctgatacct
Murine *Sqstm1* (R)	This paper	ttcctccttggctttgtctc
Murine *Cxcl1* (F)	This paper	GCTGGGATTCACCTCAAGAA
Murine *Cxcl1* (R)	This paper	CTTGGGGACACCTTTTAGCA
Murine *Cxcl2* (F)	This paper	GCCAAGGGTTGACTTCA
Murine *Cxcl2* (R)	This paper	TGTCTGGGCGCAGTG
Murine *Il6* (F)	This paper	AGTTGCCTTCTTGGGACTGA
Murine *Il6* (R)	This paper	TCCACGATTTCCCAGAGAAC
Murine *Arg1* (F)	This paper	AAGAAAAGGCCGATTCACCT
Murine *Arg1* (R)	This paper	CACCTCCTCTGCTGTCTTCC
Murine *Il1b* (F)	This paper	ACCTGCTGGTGTGTGACGTTCC
Murine *Il1b* (R)	This paper	GGGTCCGACAGCACGAGGCT
Murine *Ifng* (F)	This paper	GGCCATCAGCAACAACATAAGCgT
Murine *Ifng* (R)	This paper	TGGGTTGTTGACCTCAAACTTGGC
Murine *Il17* (F)	This paper	ATCCCTCAAAGCTCAGCGTGTC
Murine *Il17* (R)	This paper	GGGTCTTCATTGCGGTGGAGAG
Murine *Il10* (F)	This paper	CCAAGCCTTATCGGAAATGA
Murine *Il10* (R)	This paper	TCACTCTTCACCTGCTCCAC
Murine *Actb* (F)	This paper	CCAGAGCAAGAGAGGTATCC
Murine *Actb* (R)	This paper	CTGTGGTGGTGAAGCTGTAG
Recombinant DNA		
N/A	N/A	N/A
Software and Algorithms		
GraphPad Prism 8, version 8.4.3	Scientific Software	https://www.graphpad.com/scientific-software/prism/
Ingenuity Pathway Analysis	QIAGEN	https://digitalinsights.qiagen.com/?promo=qiagen-ipa
ImageJ 1.53a	National Institutes of Health	https://imagej.nih.gov

## Highlights

- Pathogenic bacteria induce DHPS and hypusination of EIF5A in macrophages
- Hypusinated EIF5A controls the translation of numerous antimicrobial effectors
- Macrophage DHPS is required to clear gastrointestinal pathogens
