# Supplementary material for: Hypusination Orchestrates the Antimicrobial Response of Macrophages
Source: Cell Rep. Author manuscript; Available in PMC 2021 Jan 18. (PMC7812972; doi:10.1016/j.celrep.2020.108510)
Supplement: 1 [file NIHMS1657017-supplement-1.pdf]

Cell Reports, Volume *33*

Supplemental Information

Hypusination Orchestrates the Antimicrobial Response of Macrophages

Alain P. Gobert, Jordan L. Finley, Yvonne L. Latour, Mohammad Asim, Thaddeus M. Smith, Thomas G. Verriere, Daniel P. Barry, Margaret M. Allaman, Alberto G. Delagado, Kristie L. Rose, M. Wade Calcutt, Kevin L. Schey, Johanna C. Sierra, M. Blanca Piazuelo, Raghavendra G. Mirmira, and Keith T. Wilson

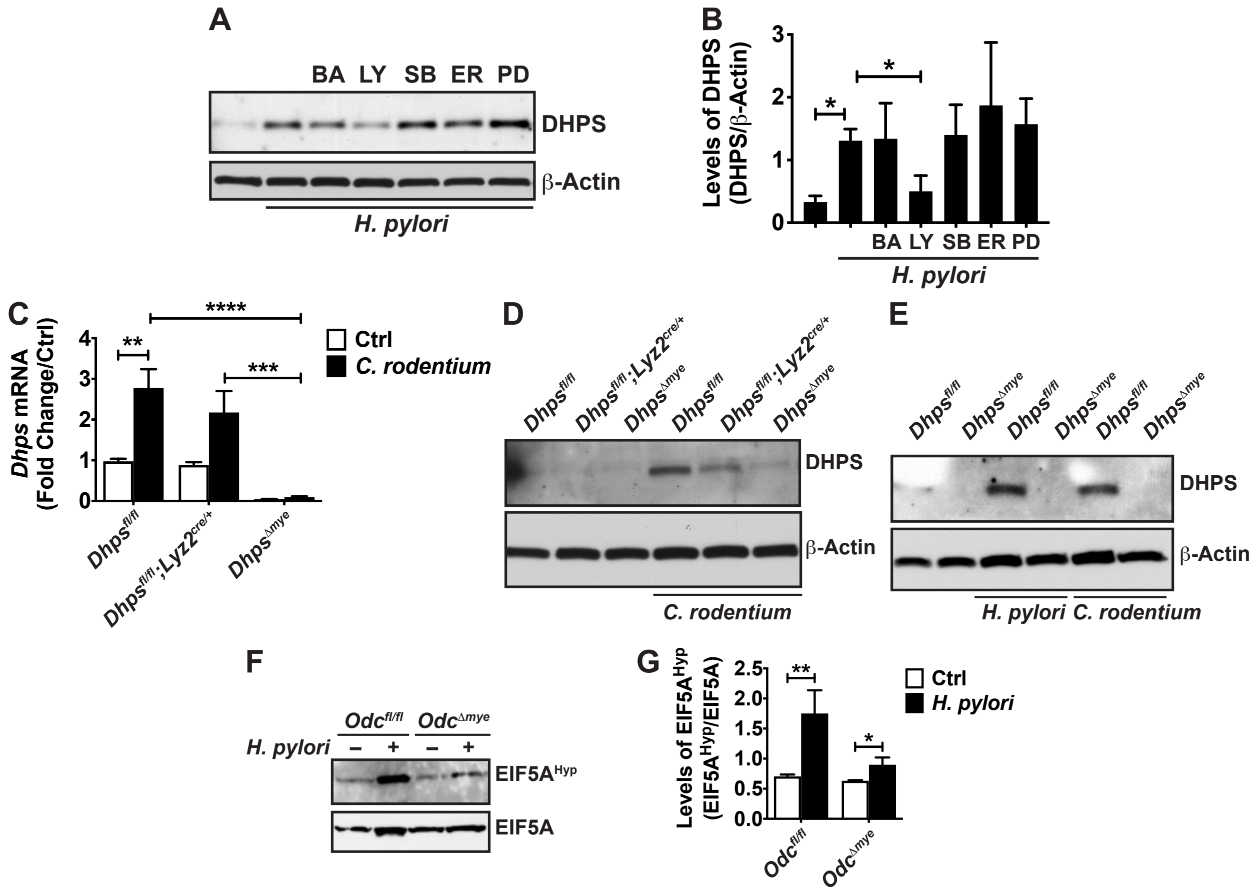

Figure S1. Analysis of DHPS induction. Related to Figure 1.

A-B, Signal transduction involved in DHPS induction. RAW 264.7 cells were pretreated or not for 30 min with Bay11-7082 (BA; 5 μM), LY294002 (LY; 10 μM), SB203580 (SB; 2 μM), ERK inhibitor (ER; 20 μM), or PD98059 (PD; 10 μM) for 30 min prior to infection with *H. pylori* PMSS1. **A,** DHPS and β-actin levels were assessed by Western blotting after 24 h. A representative blot from 2 independent experiments is shown. **B,** Densitometric analysis; *n* = 2-4. **C-E**, Effect of *C. rodentium* on DHPS expression in BMmacs and BMDCs. *Dhps* mRNA expression in BMmacs from $Dhps^{fl/fl}$, $Dhps^{fl/fl}$;$Lyz2^{cre/+}$, and $Dhps^{\Delta mye}$ mice stimulated or not with *C. rodentium* for 6 h (**C**); *n* = 3 mice per genotype. ***P*<0.01; ****P*<0.001; *****P*<0.0001. Western blot for DHPS and β-actin in the same cells infected or not with *C. rodentium* for 24 h (**D**); representative data of macrophages isolated from 3 different mice per genotype. Western blot for DHPS and β-actin in BMDCs infected or not with *H. pylori* or *C. rodentium* for 24 h (**E**); representative data of macrophages isolated from 3 different mice per genotype. **F-G**, Regulation of hypusination by ODC. BMmacs from $Odc^{fl/fl}$ and $Odc^{\Delta mye}$ macrophages were infected or not with *H. pylori* for 24 h. protein were extracted and the level of $EIF5A^{Hyp}$ and EIF5A was determined by Western blot (**F**); representative image of experiments performed with 3 mice per genotype. Densitometric analysis of the immunoblots (**G**). **P*<0.05; *n* = 3 mice per genotype.

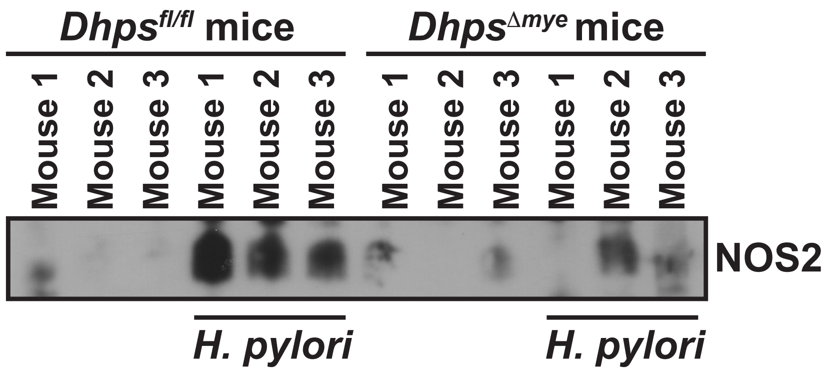

Figure S2. NOS2 translation analysis. Related to Figure 3.
BMmacs from *Dhps*$^{fl/fl}$ and *Dhp*$^{\Delta mye}$ mice (*n* = 3/genotype) were infected or not with *H. pylori* for 18 h, and Click-IT homopropargylglycine was incorporated for 4 h in a medium devoid of methionine and cysteine. Proteins were then extracted, labeled with biotin azide, and immunopurified with a NOS2 Ab. The precipitates were analyzed by Western blot using streptavidin HRP.

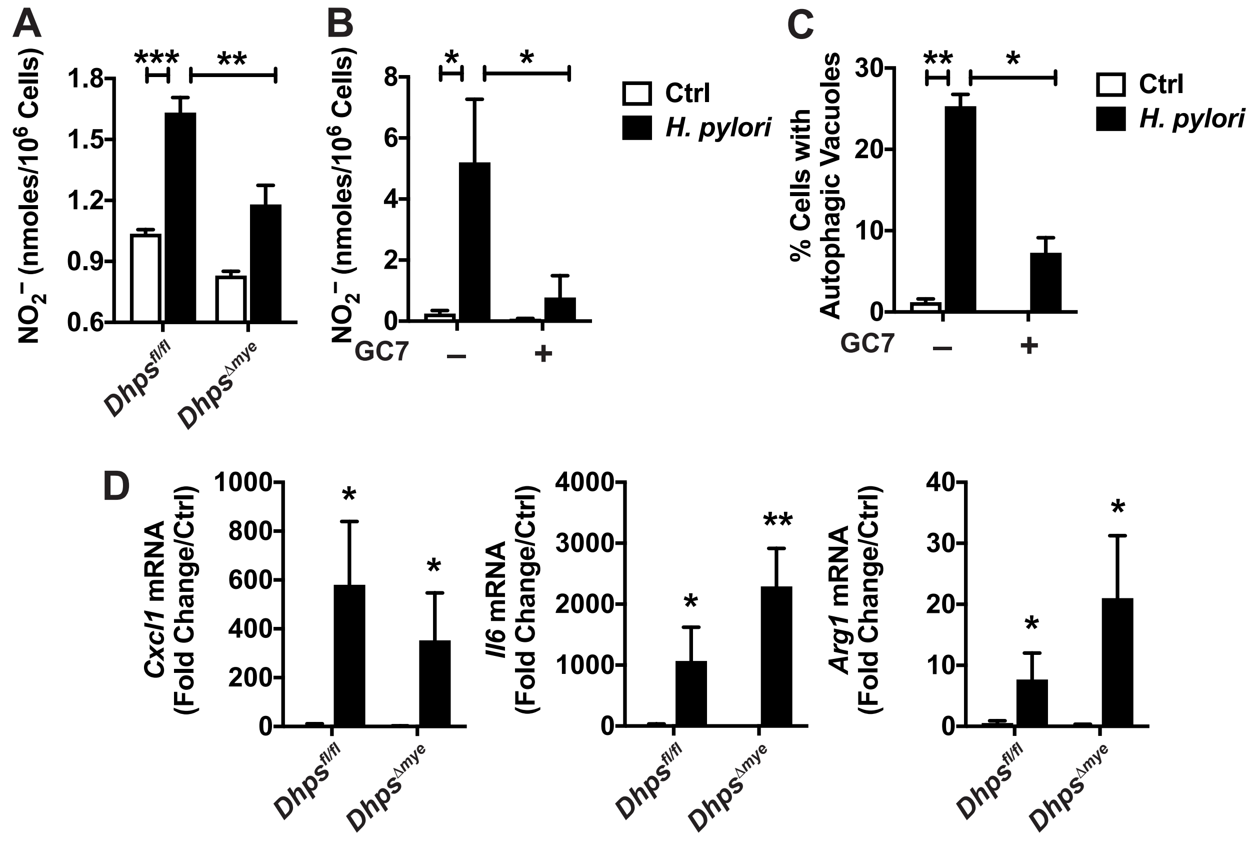

Figure S3. Effect of hypusination on NO production, autophagy, and immune gene expression. Related to Figures 3 and 4.

A-**B**, The concentration of NO_2^- was measured in the supernatant of BMmacs from *Dhps*$^{fl/fl}$ and *Dhps*$^{\Delta mye}$ mice (**A**; $n = 3$ mice/genotype) and RAW 264.7 macrophages ± GC7 (**B**; $n = 5$ independent experiments) infected or not with *H. pylori* for 24 h. **C,** Autophagy was assessed after 24 h of infection by flow cytometry. In all the panels, *$P<0.05$; **$P<0.01$; ***$P<0.001$. **D**, The expression of the genes encoding for CXCL1, IL-6, and arginase-1 was analyzed in BMmacs from *Dhps*$^{fl/fl}$ ($n = 3$) and *Dhps*$^{\Delta mye}$ mice ($n = 3$) infected or not with *H. pylori* PMSS1 for 6 h. In all the panels, *$P<0.05$; **$P<0.01$.

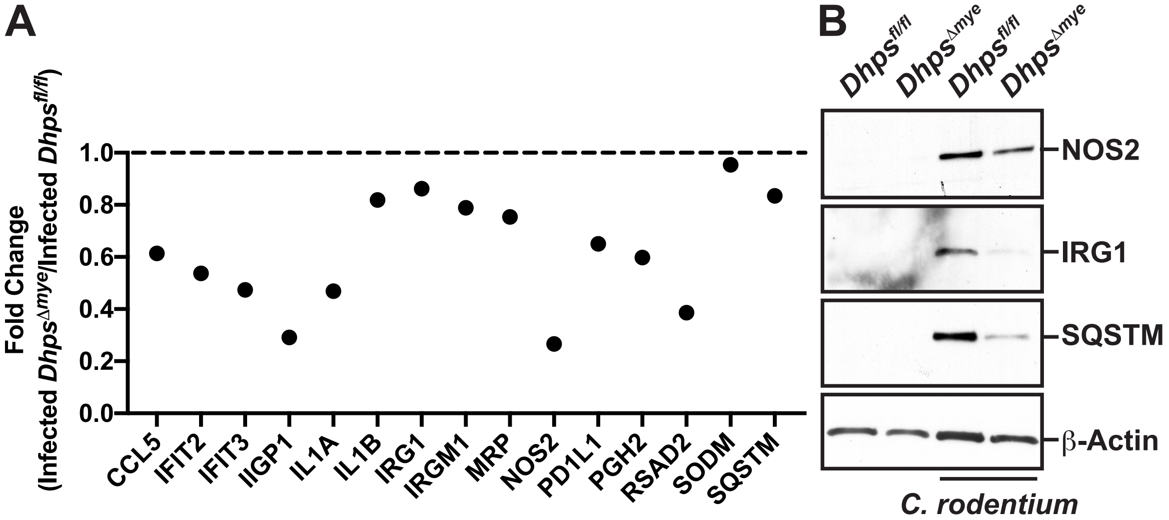

Figure S4. Change in protein expression in macrophages with *Dhps* deletion. Related to Figure 3.
A, BMmacs from *Dhps*$^{fl/fl}$ and *Dhps*$^{\Delta mye}$ mice (one of each genotype) were infected with *H. pylori* for 24 h, and their proteomes were analyzed by iTRAQ (see Supplementary Table 2). Only the inducible proteins regulated by *Dhps* deletion in the first proteomics analysis (see Fig. 3) are depicted in this panel. **B**, BMmacs from *Dhps*$^{fl/fl}$ and *Dhps*$^{\Delta mye}$ mice were infected or not with *C. rodentium*. Levels of NOS2, IRG1, SQSTM, and β-actin proteins were analyzed by Western blot. This panel is a representative of the results obtained with 2 mice per genotype.

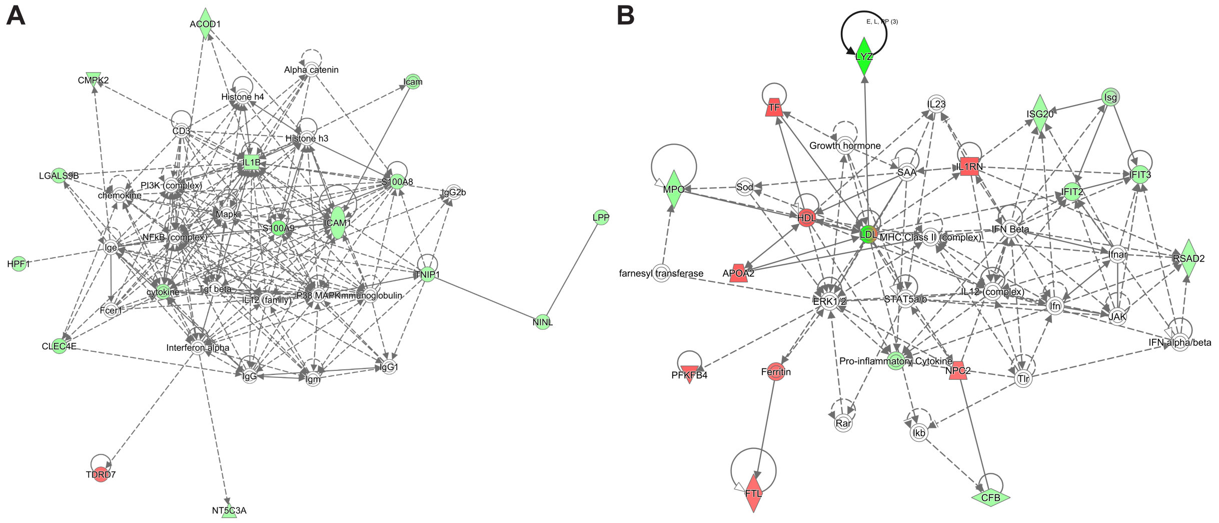

Figure S5. Protein-Protein interaction networks identified by IPA of the proteome of *H. pylori*-infected BMmacs from *Dhps*$^{\Delta mye}$ mice compared to *Dhps*$^{fl/fl}$ mice. Related to Figure 3. **A**, Hematological System Development and Function, Inflammatory Response, Tissue Morphology. **B**, Antimicrobial Response, Connective Tissue Disorders, Inflammatory Response.

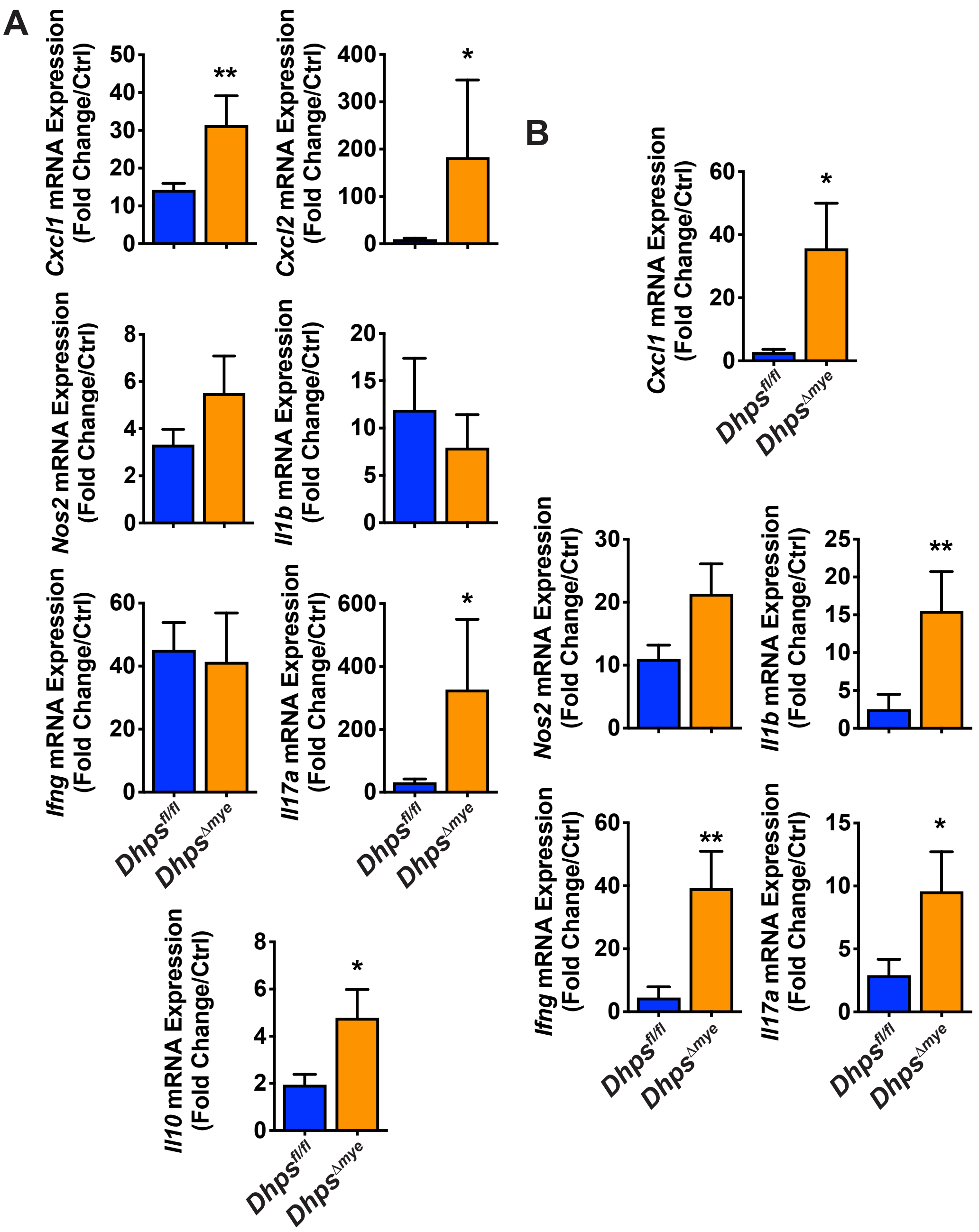

Figure S6. Expression of the genes encoding for chemokines and cytokines. Related to Figures 5 and 6.
mRNA expression was analyzed by RT-real-time PCR from gastric (**A**) and colon (**B**) tissues from *Dhps*$^{fl/fl}$ and *Dhps*$^{\Delta mye}$ mice infected with *H. pylori* (8 weeks) or *C. rodentium*, respectively. **P*<0.05; ***P*<0.01; *n*=5-9 mice per group.

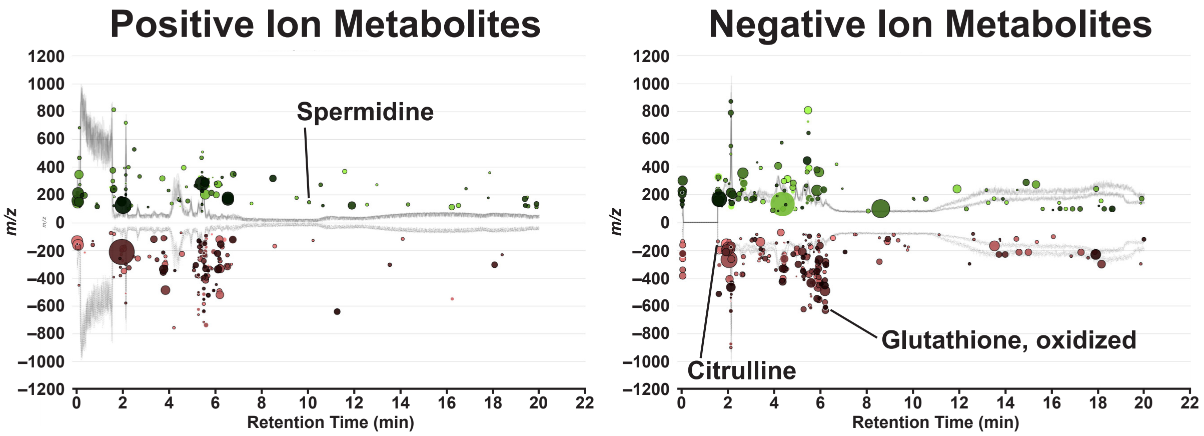

Figure S7. Differential gastric metabolome of *H. pylori*-infected *Dhps*$^{\Delta mye}$ mice compared to *Dhps*$^{fl/fl}$ animals. Related to Figure 5.
A metabolomic analysis was performed on gastric tissues from *Dhps*$^{fl/fl}$ and *Dhps*$^{\Delta mye}$ mice infected or not with *H. pylori*. Cloud plots for the metabolites downregulated (red) and upregulated (green) by more than 1.3-fold (P<0.05) were generated using Xcms software https://xcmsonline.scripps.edu). The retention time and the mass-to-charge ratio is represented by the position on the x-axis and y-axis, respectively. The fold change of each metabolite is represented by the radius of each dot and the P-value is represented by how dark or light the color is.
