# Supplementary material for: Hypusination Orchestrates the Antimicrobial Response of Macrophages
Source: Cell Rep. Author manuscript; Available in PMC 2021 Jan 18. (PMC7812972; doi:10.1016/j.celrep.2020.108510)
Supplement: 7 [file NIHMS1657017-supplement-7.docx]

**Table S6.** Presence of the AAAUGU consensus in the mRNA sequences and frequencies of EIF5A^Hyp^-regulated peptides in the proteins identified to be infected by hypusination in BMmacs or RAW 264.7 macrophages.

| **Proteins** | **nnnnnAAAUGUnnnnn sequence** | **Number of**  **diprolyl motifs** | **Number of**  **diglycyl motifs** | **EIF5A-regulated motifs^1^** |
| --- | --- | --- | --- | --- |
| ACSL1 | GGGGCAAAUGUGUUCA | 2 | 1 | PPE |
| ALD2 | None | 1 | 0 | SPP |
| ASS1 | GGCCGAAAUGAUCUGA  GUGAUAAAUUUGUAAU | 1 | 1 | PPG |
| B2CL1 | UAUAAAAAUGUCUCAG | 0 | 2 | None |
| BST2 | GCUCAAAGUGUCACUG | 0 | 1 | None |
| CAV1 | UGACAAAAUAUUGGUU  UGACAAAAUAUUGGUU | 0 | 1 | None |
| CCL5 | AGCAGCAAGUGCUCCA  ACCGCCAAGUGUGUGC  GAGAAGAAGUGGGUUC | 0 | 0 | None |
| CCL9 | AAUUGAAAUGUUUCAC | 1 | 0 | None |
| CD14 | UACAUAAAUUUACAGG  CUUGAAAAUCUCGAGG  GCUUUAAAUUUAUUAA  UAUUAAAAUCUUAAUC | 2 | 1 | APP/GGG/PPP |
| CLEC4E | CUCUCAAAUUUCCGGG  AGAGUGAAUGUAAUAA  AAGUAAAAUGACUUGU  CUAACAAAUGCAACAA | 0 | 0 | None |
| CMPK2 | AGUUCAAAUGUUGAUG | 3 | 1 | PPG/PPP/PPA |
| DERPC | None | 4 | 11 | PPR/PGG/PDP/GPP/PGG/PGG |
| EHD1 | GUCUGAAAUGUAGCCC | 2 | 1 | None |
| FABPH | None | 0 | 1 | None |
| GBP2 | AAGACAAAUGUCUUGC | 0 | 2 | PGG |
| GBP4 | ACCACAAAUGGCUCCC  AAGUUAAAUGGGGAAG  CAAGGAAAUGAAACAA  AAACAAAAUGGUGAUU  CACAGAAAUGAGAUAG | 0 | 2 | PGG |
| GLRA1 | CUGGAAGAUGUACAGC  AAUGGCAAUGUCCUCU  GAUCCAGAUGUACAUC  CUUCAACAUGUUCUAC | 2 | 1 | GPP/PPP |
| ICAM1 | GAAGGAAAUGUUCCAA | 2 | 4 | PPP |
| IFIH1 | CAAGAAAAUGUUGAAA  ACCAAAAAUGUAAACA  UUGGUAAAUGUAAUUG | 0 | 1 | GGG/DDG |
| IFIT2 | GAAUUAAAUGUGUAAA  UUUGAAAAUGUCAUGG  AUGUGAAAUGUGUCAC  GGCCUAAAUGUUAAGG  UAAAAAAAUGUUUCUC | 1 | 1 | PPG/GGG |
| IFIT3 | AGCAAAAAUGUACUUU | 0 | 0 | None |
| IIGP1 | AACAAAAAUGUUUGUC | 2 | 0 | EPP |
| IKKE | UGUCUAAAUGUUCCCA | 1 | 2 | PPE/PDP |
| IL1A | AGUGAAAAUGAAGACU  UGGUUAAAUGACCUGC  AUUCUAAAUGAAUGUU  CUUCAAAAUGCCAGUU  AUCUAAAAUGAUAAUU | 0 | 1 | None |
| IL1B | GCUUCAAAUCUCACAG | 1 | 0 | PPF |
| IRG1 | UAGUAAAAUCUACAGU | 4 | 0 | PPD |
| IRGM1 | CUAUUAAAUGUGAUAU | 2 | 0 | PPV/PPQ |
| ISG20 | CUACAAAAUCUCUCAG  CACCGCAAUGUGUAAA | 1 | 2 | None |
| ITA5 | AAGAAACAUGUGUACC  GCCCAAAAUCUGGGUG | 4 | 11 | PPP/GPP/PPG/RDK/PPA |
| LIPB2 | GACAGAAAUGUGAUGC  GCAUCAAAUGUGCUAU | 4 | 3 | SPP/PPP/SPP/PPQ/RDK |
| MTDC | GUUUAAAAUGUUUGUA | 0 | 2 | PGG/DVG/PGG |
| MRP | UUUAUAAAUGUCUUAC  GGAAUAAAUGUCGUCC | 2 | 1 | PPV/APP/GGG |
| NAA40 | GGGUUGAAUGUCUCCA  CCAUUGAAUGUAAGCG | 0 | 2 | None |
| NINL | GGAUGAAAUGUCACAG  AACAAAAAUGUUGUCA | 3 | 0 | EPP/DDG/RDK/DVG/RDK |
| NOS2 | ACAGGAAAUGUUUCAG | 4 | 3 | DDP/PPD/PPV |
| OAS1 | GCCGUCAAUGUCGUGU  UCGAGGAAUGUACCUC | 1 | 2 | PDP/PPQ |
| PGH2 | GGAAGAAAUGUGCCAA | 4 | 3 | SPP/PPV/PDP |
| PLD3 | None | 1 | 1 | PPP/DDG |
| RREB1 | CAGGAAAAUGUUUCCU  AUCCCAAAUGUAGAUU  GCAAUAAAUGUUAUAU | 12 | 4 | PPG/APP/PPQ/PPP/PPQ/PPP/PPP/PPA |
| RSAD2 | ACUACAAAUGUGGCUU  AUAUAAAAUGUGGCAG  GGUAUAAAUGUGCCUG | 0 | 3 | DVG |
| SDCB1 | UGCAGAAAUGUCUCUU  UUAUUAAAUGUAUAAA  UAUAAAAAUGUGAAAU | 1 | 0 | PPD |
| SODM | UGGGAGAAUGUUACUG | 0 | 2 | GGG/GGG |
| SQSTM | AGAGCAAAUGAAAAAG | 6 | 4 | PGG/PPG/PPR/APP/PPE/PPP |
| SRPRA | UUCCAAAAUGACUUCC  GCUAAGAAUGUGGCUG  AGGCUUAAUGUGGCUC | 1 | 6 | RDK/PGG/PPE |
| STAP1 | UGCCUGAAUGUUUUUA  UGGGGAAAUAUGAUCC | 2 | 0 | PPE |
| STAT2 | AGUCCAAAUGGCGCAG  GUGGGAGAUGUUGCAG  GGGUGACAUGUUUGCC  GAGUUACAUGUCAUCA  GAAGAAGAUGUUGUCU  AAACCCAAUGUUGGCU  CAUCAAGAUGUUGCUU  GACUUUAAUGUUUUGA | 3 | 1 | PPP/PPP/PPG |
| TNFA | GCCUCGAAUGUCCAUU | 0 | 2 | None |
| UPP1 | None | 0 | 2 | None |
